# The cell cycle, autophagy, and cell wall integrity pathway jointly governed by MoSwe1 in *Magnaporthe oryzae*

**DOI:** 10.1186/s12964-023-01389-6

**Published:** 2024-01-09

**Authors:** Lin Li, Xue-Ming Zhu, Jian-Dong Bao, Jiao-Yu Wang, Xiao-Hong Liu, Fu-Cheng Lin

**Affiliations:** 1 State Key Laboratory for Managing Biotic and Chemical Treats to the Quality and Safety of Agro-products, Institute of Plant Protection and Microbiology, Zhejiang Academy of Agricultural Sciences, Hangzhou, 310021 China; 2 Institute of Biotechnology, Zhejiang University, Hangzhou, 310058 China

**Keywords:** Cell cycle, Autophagy, CWI pathway, Appressorium formation, Virulence, *M. Oryzae*

## Abstract

The cell cycle is pivotal to cellular differentiation in plant pathogenic fungi. Cell wall integrity (CWI) signaling plays an essential role in coping with cell wall stress. Autophagy is a degradation process in which cells decompose their components to recover macromolecules and provide energy under stress conditions. However, the specific association between cell cycle, autophagy and CWI pathway remains unclear in model pathogenic fungi *Magnaporthe oryzae*. Here, we have identified MoSwe1 as the conserved component of the cell cycle in the rice blast fungus. We have found that MoSwe1 targets MoMps1, a conserved critical MAP kinase of the CWI pathway, through protein phosphorylation that positively regulates CWI signaling. The CWI pathway is abnormal in the Δ*Moswe1* mutant with cell cycle arrest. In addition, we provided evidence that MoSwe1 positively regulates autophagy by interacting with MoAtg17 and MoAtg18, the core autophagy proteins. Moreover, the S phase initiation was earlier, the morphology of conidia and appressoria was abnormal, and septum formation and glycogen degradation were impaired in the Δ*Moswe1* mutant. Our research defines that *MoSWE1* regulation of G1/S transition, CWI pathway, and autophagy supports its specific requirement for appressorium development and virulence in plant pathogenic fungi.

Video Abstract

**Supplementary Information:**

The online version contains supplementary material available at 10.1186/s12964-023-01389-6.

## Introduction

*Magnaporthe oryzae* differentiates specialized infection structures-appressoria to breach the plant cuticle, which is the causal agent of rice blast disease in cereal plants [46, 56, 64, 75]. During the appressoria maturation, the nuclei of conidia are degraded via the autophagy pathway; then, the overall conidia contents are transported to appressoria [68]. In the process of continuous accumulation of turgor pressure to the maximum 8 MPa [24], the appressoria dynamically remodel their actin cytoskeleton, thereby forming a circular F-actin network at the base of the appressorium. Septin GTPases support F-actin and form a penetrating peg at the base of the appressorium to penetrate plant leaves [11]. When the three-cell conidia germinate, the apical cells undergo only one round of mitosis. One nucleus divides to form two nuclei, one entering the appressorium and the other returning to the apical cell. The basal cell and the middle cell do not undergo mitosis and septum formation. When the appressorium matures, each nucleus is degraded by autophagy, resulting in cell death of the conidia and recycling of the conidia bulks into the appressoria [28, 52].

The eukaryotic cell cycle is a highly coordinated and conserved process. The cell cycle and cell division cooperate to regulate fungal morphogenesis, including the stage of conidial germination and the location of appressorial penetration of plants [15, 61]. In *Colletotrichum orbiculare*, appressorium formation is required for suitable cell cycle progression, which CoBub2 and CoBfa1 regulate. Disruption mutants initiate earlier G1/S progression during conidial germination [16]. In *M. oryzae*, the ability of appressoria to infect plants is closely related to cell cycle-mediated regulation [53, 62]. Appressoria development and the emergence of penetration pegs depend on the synthesis phase (S phase) of the cell cycle [52, 61]. Recent studies have shown that rice blast infection is controlled by two independent S-phase checkpoints operating in two successive cell cycles. The first checkpoint occurs during the initial stages of appressoria formation and is related to the DNA damage response (DDR) pathway, with MoCds1 kinase involvement. The second checkpoint is controlled in a completely different way by the turgor pressure of the appressorium, which controls NADPH oxidase activation and the remodeling of septin-dependent F-actin at the base of appressoria. This process regulates the emergence of the penetration peg and leads to plant infection [53].

The Mitogen-activated protein kinase (MAPK) cascades, ubiquitous in all eukaryotes, include three-tiered protein kinases modules that sequentially transmit various cellular signals [74]. In *Saccharomyces cerevisiae*, the CWI pathway regulates cell wall remodeling under various stresses by coordinating cell wall biosynthesis and actin organization, as well as other events that maintain cell integrity [32]. The CWI pathway is activated by cell surface sensors such as Slg1/Wsc1, Wcs2, Wsc3, Mid2, and Mtl1. These sensors are coupled to the small G protein Rho1 under cell wall stress signals. Rho1 then progressively activates the protein kinase C (Pkc1), upstream kinase MAP kinase kinase (Bck1), a pair of redundant MAP kinase kinases (Mkk1 and Mkk2), and MAP kinase (Slt2/Mpk1) [31, 45]. This cascade regulates nuclear localization, thereby activating the activity of transcription factors Rlm1 and SBF complexes (Swi4 and Swi6) to regulate the expression of cell wall biosynthesis genes [26, 32]. In *M. oryzae*, the CWI pathway MoRho1 (Rho1 homolog), MoPkc1 (Pkc1 homolog), MoMck1 (Bck1 homolog), MoMkk1(Mkk1/2 homolog), and MoMps1 (Mpk1/Slt2 homolog) play an important role in appressorium formation, appressorium penetration, and pathogenicity [25, 77, 78].

Autophagy is a conserved cellular process that engulfs cargo into vesicles and transports them into vacuoles to degrade and recycle cargo [76]. The nuclear degeneration of conidia and appressoria in *M. oryzae* occurs through macroautophagy. Any deleting mutant a core autophagy gene exhibits a loss of pathogenicity [28], suggesting that autophagy is necessary for pathogenic fungi to infect plants. In *M. oryzae*, autophagy pathway is composed of a MoAtg1-MoAtg13-MoAtg17 complex, a MoAtg9 trafficking system, MoAtg6-MoAtg14(MoVps38)-MoVps34-MoVps15 complex, Atg8-PE ubiquitin and the MoAtg12-MoAtg5-MoAtg16 ubiquitin-like systems [37, 49, 65, 70]. MoAtg1-MoAtg13-MoAtg17 affects the formation of autophagosomes. External stimuli (ROS or starvation or rapamycin) inhibit TOR activity, dephosphorylate MoAtg13 and bind to MoAtg17, activating MoAtg1 and marking the initiation of autophagy [35]. MoAtg18 interacts with MoAtg2 and MoAtg9 to influence the positioning of Atg9 [69].

In *S. cerevisiae*, Swe1 (*S*. *cerevisiae*wee1 homolog) inhibits the activity of Cdk1/Cdc28-Clb2 through phosphorylation of Y19 (the 19th amino acid site of tyrosine) of Cdk1 [7]. This modification process can be reversed by dephosphorylation of Mih1 [59]. Swe1 controls the timing of entry into mitosis in *S. cerevisiae* [27]. Swe1 appears for the first time in the nucleus of the late G1 phase. Swe1 is localized to the daughter bud side of the mother bud neck in a form-dependent Hsl1 kinase and Hsl7 protein when the bud appears [39]. Before late G2 is degraded, Swe1 in the bud neck is hyperphosphorylated by Cdc5, Cla4, and Cdk1-Clb2 proteins [1, 21, 23, 48, 60]. The hyperphosphorylated form of Swe1 is susceptible to ubiquitination and can be degraded by the 26 S proteasome [48]. In *S. cerevisiae*, the primary function of the three kinase proteins (Hsl1, Kcc4, Gin4) is to transfer Swe1 to the bud neck, facilitating Swe1 degradation and the subsequent cell cycle. Moreover, the functions of the three kinase proteins are different [41, 50]. Gin4 and Kcc4 cooperate with the PAK kinase Cla4 to participate in the reorganization of septin, and Hsl1 is directly involved in the degradation progression of Swe1 [3, 9]. In *M. oryzae*, Gin4 (MGG_02810) is a homologous protein of Nim-related kinase and arginine N-methyltransferase Hsl7 (MGG_03894) localized in appressoria pores before the infection of plants [52]. However, how the mitosis progression is regulated remains unclear.

In this study, we demonstrated the functions of MoSwe1 in conidium, appressorial morphogenesis, appressorial differentiation, septum formation, and plant invasion in *M. oryzae*. Our results found that MoSwe1 positively regulates the CWI pathway by affecting MoMps1 phosphorylation levels. In addition, MoSwe1 interacts with autophagy proteins MoAtg17 and MoAtg18 to regulate autophagy. We report here a novel link, namely MoSwe1 linkage of cell cycle control, CWI signaling, and autophagy, that underlies the growth and pathogenicity of rice blast fungus.

## Results

### Identification of Swe1 in *M. oryzae*

To explore the functions of Swe1 in plant pathogenic fungi, we acquired the sequences of MoSwe1 in *M. oryzae*. Taking the *S. cerevisiae* Swe1 protein as a query, MGG_01816 was identified as the homolog of the Wee protein kinase Swe1 through a search of the *M. oryzae* genome database EnsembIFungi (http://fungi.ensembl.org/Magnaporthe_oryzae/Info/Index?db=core). MGG_01816 shows 30.62% identity with ScSwe1. Thus, MGG_01816 was named MoSwe1. We cloned the full-length Swe1 cDNA sequence, which encodes an 1110 amino acid protein in *M. oryzae*. Sequence alignment supported that the Swe1 protein is well conserved among eukaryotes (Fig. S1A). The results showed that the MoSwe1 protein shares 44.01% amino acid identity with the homologous protein in *C. orbiculare*, 42.41% identity with the homologous protein in *Fusarium graminearum*, 38.03% identify with that in *Aspergillus fumigatus*, 43.03% identify with that in *Fusarium oxysporum*, 32.08% identify with that in *Mus musculus*, and 32.27% with that in *Homo sapiens*. We predicted the protein structure of Swe1 by AlphaFold2. We found that Swe1 had conserved regions in *M. oryzae* (Fig. S1B), consistent with the amino acid sequence alignment results shown in Fig. S1A.

### Swe1 functions in G1/S progression by regulating Cdc28 phosphorylation

**Fig. 1:**
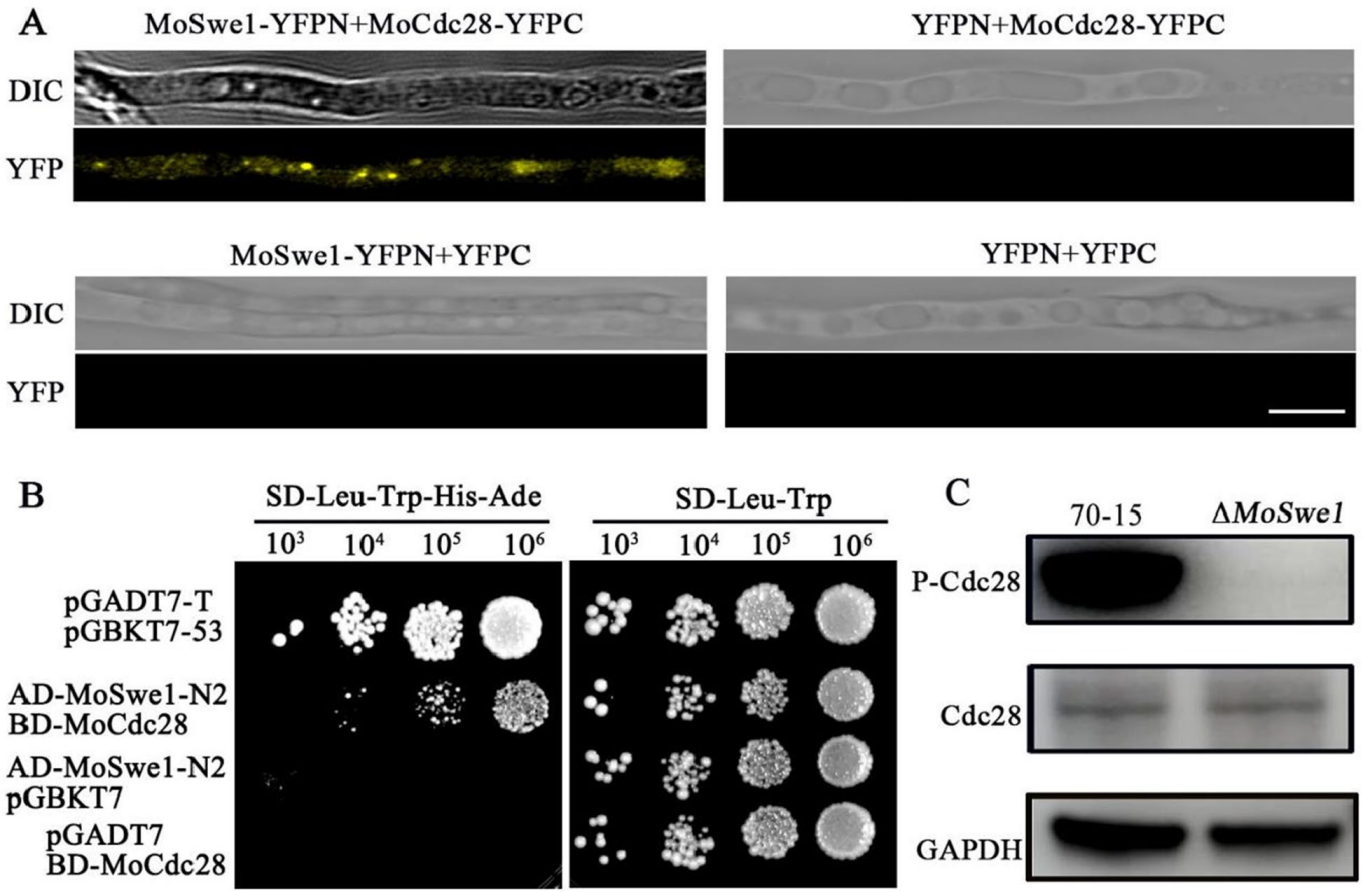
MoSwe1 interacts with MoCdc28 to affect its phosphorylation level. **A** BiFC was used to detect the interaction relationship between MoSwe1 and MoCdc28. The YFP signals can be observed in MoSwe1-YFPN and MoCdc28-YFPC co-transformed strains: scale bar, 5 μm. **B** Yeast two-hybrid analysis to examine the interaction of MoSwe1-N2 and MoCdc28 in vivo. The positive control was pGBKT7-53 and PGADT7-T. Pairs of pGBKT7 and AD-MoSwe1-N2 and pGADT7 and BD-MoCdc28 were negative controls. **C** Detection of the Cdc28 phosphorylation in the Δ*Moswe1* mutant. Western blot was carried out to detect Cdc28 Y15 (for phosphorylation signal) and Cdc28 in protein extracts using the anti-phospho-Cdc28 (Tyr15) and anti-Cdc28 antibodies, respectively

To characterize the functions of MoSwe1, we replaced the Swe1 protein coding sequence with a glufosinate-ammonium resistance gene (*BAR*) to produce Δ*Moswe1* deletion mutant (Fig. S2). The Swe1/Wee1 kinase phosphorylates and inhibits Cdk1(Cdc28)-Clb2, a primary mitotic switch in yeast [59]. To determine whether MoSwe1 regulates the Cdc28 phosphorylation and cell cycle progression, we demonstrated the interaction between MoSwe1 and MoCdc28 through yeast two-hybrid system and bimolecular fluorescence complementation (BiFC) experiments (Fig. 1A and B). We used P-cdc2 (Tyr15) antibody to study MoCdc28 (Cdc2 homolog) phosphorylation in the wild type 70 -15 and the Δ*Moswe1* mutant and found that MoCdc28 phosphorylation levels were lost entirely in the Δ*Moswe1* mutant (Fig. 1C). Data from the quantitative phosphoproteomic data also showed a complete loss of MoCdc28 (Tyr15) phosphorylation in the Δ*Moswe1* mutant (Fig. 8F). These results indicated that MoSwe1 affects the phosphorylation of MoCdc28.

It is well known that the S phase of mitosis carries out DNA replication and synthesis, controlling initial appressorial development and the emergence of penetration pegs in many pathogenic fungi [53, 62]. When a particular concentration of DNA replication inhibitor Hydroxyurea (HU) is added to the germinating conidia, initial appressoria cannot be formed. In addition, when HU was used to treat mature appressoria, the formation of penetration pegs and plant infection were blocked in *M. oryzae* [53, 62]. To further explore the specific mitotic processes (G1 phase, S phase, G2 phase, M phase) affected by MoSwe1, we collected conidia of the 70-15, Δ*Moswe1* and Δ*Moswe1-C* strains to induce appressoria on hydrophobic coverslips. HU was added to the Δ*Moswe1* mutant at 2 h post-incubation (hpi), and almost no appressoria had formed at 24 hpi (Fig. S3A and B). The phenotype of the Δ*Moswe1* mutant was consistent with that of the 70-15. The data showed that the Δ*Moswe1* mutant is not sensitive to HU and that MoSwe1 does not directly participate in determining the S phase of the cell cycle.

**Fig. 2:**
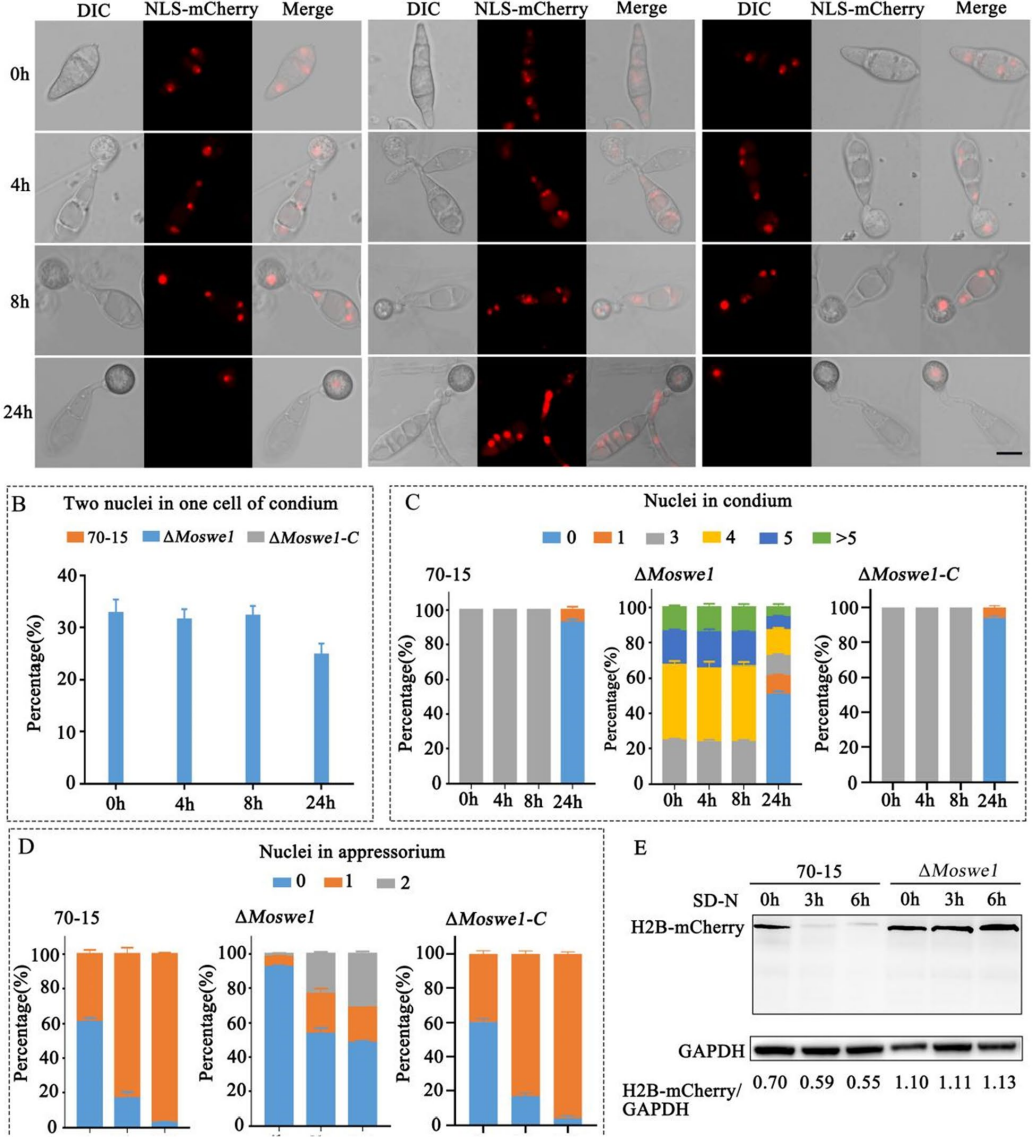
MoSwe1 functions in the cell cycle and regulates G1/S progression in *M. oryzae*. **A** Visualization of nuclei during appressorium development using NLS-mCherry. The merged image shows NLS-mCherry and DIC: bars, 10 μm. **B** The number of occurrences of two nuclei in one cell of the conidium during conidial germination. **C** The number of nuclei in conidia during conidial germination. **D** The number of nuclei in appressoria during conidial germination. **E** Degradation of H2B-mCherry was examined in 70-15 and Δ*Moswe1*. The 70-15 and Δ*Moswe1* strains with H2B-mCherry tags were induced in SD-N medium for 0, 3, and 6 h, respectively. The degradation level of mCherry was detected by western blotting using mCherry antibodies

To study whether the nuclear division was affected in the Δ*Moswe1* mutant during conidial germination, red fluorescent nuclear localization proteins (NLS-mCherry) were used to observe dynamic changes in the nucleus in the 70-15 and the Δ*Moswe1* mutant. We first observed differences in nuclei during conidial germination. Surprisingly, in the Δ*Moswe1* mutant, two nuclei appeared in the basal cells and the middle cells of conidia during conidia germination (Fig. 2A). In contrast, in the wild type 70-15, nuclear division occurs only in apical cells. Nuclear division in the Δ*Moswe1* mutant occurs in the apical cells and the middle and basal cells, resulting in binucleate cells. To more accurately and intuitively understand the difference between cell nuclear division in the Δ*Moswe1* mutant and wild type 70-15, we counted the nuclei in a conidium, in one cell, and in an appressorium at different periods of conidial germination (Fig. 2B). The statistical data showed that the proportion of cells with two nuclei in a conidium during conidium germination was more than 20%. Still, there was only one nucleus in one cell in a conidium in the wild type 70-15 and Δ*Moswe1-C* complemented strain (Δ*Moswe1-C*) (Fig. 2B). In addition, during conidial germination and appressorium formation, the number of nuclei in the conidium and appressorium were counted. At 0 hpi, 4 hpi, and 8 hpi, respectively, the proportion of conidia containing more than three nuclei was more than 70% in the Δ*Moswe1* mutant compared with the 70-15 and Δ*Moswe1-C* strains, whose conidia did not have more than three nuclei (Fig. 2C). The proportion of appressoria with two nuclei in the Δ*Moswe1* mutant was more than 20% at 8 hpi and 24 hpi, while there were no binucleate appressoria in the wild type 70-15 and Δ*Moswe1-C* (Fig. 2D). We also measured the level of nucleus degradation at protein levels. mCherry antibody was used to detect the degradation level of nuclear protein H2B. We found that H2B-mCherry was always present in the Δ*Moswe1* mutant (Fig. 2E). This indicated that the cell nuclei in the Δ*Moswe1* mutant did not degrade.

**Fig. 3:**
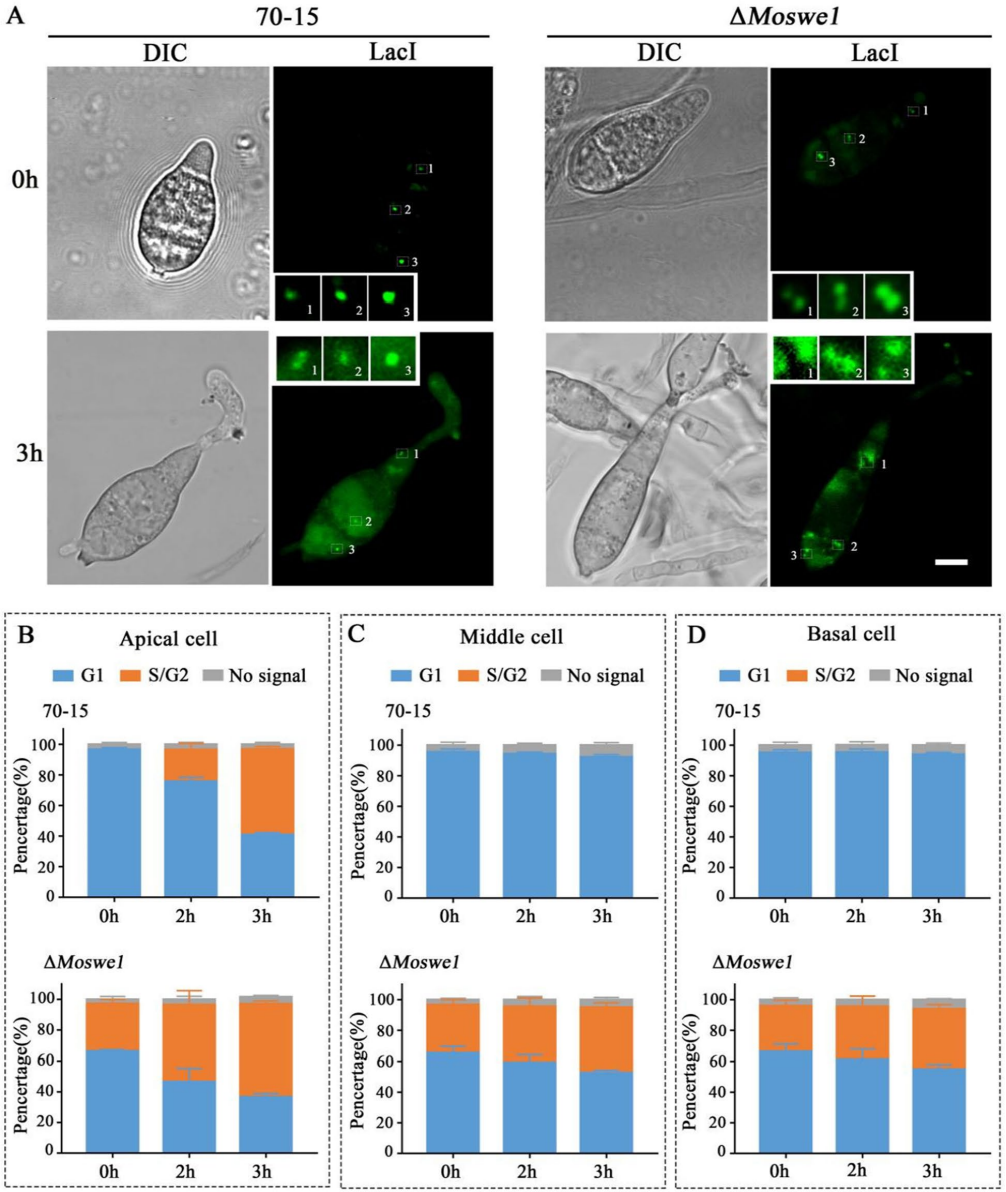
Progression of the cell cycle during conidia germination in *M. oryzae*. **A **Pictures of LacO/LacI-GFP-transformed strains of wild type 70-15 and the Δ*Moswe1* mutant during conidial germination at 0 and 3 hpi. Bars, 5 μm. **B**-**D** Mean percentage of cell cycle progression in wild-type 70-15 and Δ*Moswe1* mutant cells that showed two patterns of GFP-LacI spots in the apical (**B**), middle (**C**), and basal cells (**D**) of the conidium at 3 hpi. At least 60 conidia were scored in each cell at each time point

Next, we further examined the timing of cells entering the S phase during conidial germination to determine whether earlier nuclear division in the Δ*Moswe1* mutant was due to earlier progression in the cell cycle G1/S transition. We introduced chromosome tagging into the wild type 70-15 and the Δ*Moswe1* mutant. Yasuyuki Kubo’s group developed a method to detect the specific phase of mitosis by constructing a plasmid consisting of an array of 256 Lac operator repeats inserted into the locus of an individual chromosome in the genome and GFP-LacI-NLS fusion sequences. After the plasmid is transformed into the target strain, only one GFP point indicates that the cell is in the prereplication stage (G1). If two GFP spots are present, the cell is in the S/G2 phase, and these two spots are located on the sister chromatids [17]. Using this method, we observed the cell cycle progression of the basal cells, middle cells, and apical cells of the conidia and the cells of the appressoria in *M. oryzae*. At 0 hpi, two GFP spots were present in the middle and basal cells of the Δ*Moswe1* mutant, whereas only one GFP spot was present in all wild type 70-15 cells. The results indicated that the middle and basal cells of the Δ*Moswe1* mutant had entered the S phase (Fig. 3A). At 3 hpi, only the apical cells had two GFP spots in wild type 70-15 (Fig. 3A), indicating that only the apical cells entered S phase.

In contrast, the basal and middle cells did not enter the S phase. However, in the Δ*Moswe1* mutant, the apical cells and the middle and basal cells contained two GFP spots (Fig. 3B and D). At 24 hpi, the appressoria matured, and 30% of the appressoria of the Δ*Moswe1* mutant had four GFP spots, indicating that the nucleus in the Δ*Moswe1* mutant entered a second round of the S phase (Fig. S3C and D). Overall, the findings indicated that the G1 arrest was released immediately after the cells entered the G1 phase in the Δ*Moswe1* mutant. In other words, knocking out Mo*SWE1* affects the G1/S phases of the cell cycle by regulating MoCdc28 phosphorylation of *M. oryzae*.

### *MoSWE1* affected vegetative growth, conidiogenesis, conidial and appressorial morphology, and appressorium formation of *M. oryzae*

**Fig. 4:**
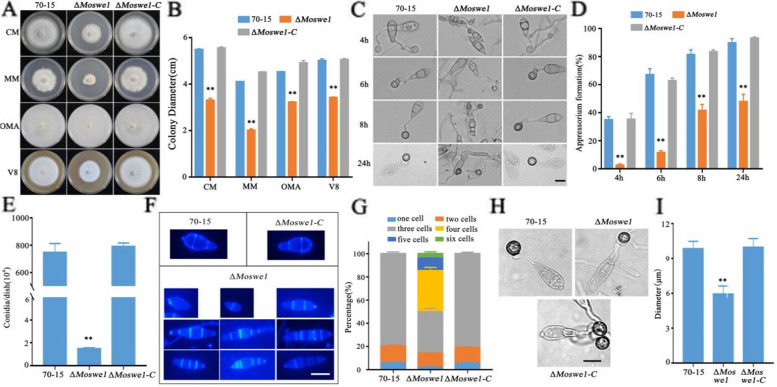
MoSwe1 is indispensable for fungal vegetative growth, appressorium formation, conidiogenesis, conidial production, and appressorial morphology in *M. oryzae*. **A** 70-15, Δ*Moswe1* and Δ*Moswe1-C* strains were grown on CM, MM, OMA, and V8 medium for 8 days. **B** Diameters of colonies. Duncan’s test (**, *P* < 0.01). **C** Appressorium formation was induced by a hydrophobic surface: scale bar, 10 μm. **D** Compute appressorium formation rate and perform statistical analysis. Asterisks delegate significant differences (*P* < 0.01). **E** Statistical analysis of conidial production. **F** Stained conidial septa of 70-15, Δ*Moswe1* and Δ*Moswe1*-*C* with CFW. Bars, 10 μm. **G** Number of conidial cells in 70-15, Δ*Moswe1* and Δ*Moswe1*-*C*. **H** Images of the appressorium were taken using a light microscope. Samples were incubated to induce appressoria in the 70-15, Δ*Moswe1* mutant and Δ*Moswe1*-*C* strains: scale bar, 10 μm. **I** Measurement of the diameter of the appressorium. The experiment was repeated three times with three replicates per treatment. Significant differences were calculated using Duncan’s test. Asterisks denote statistical significance (*P* < 0.01)

To determine whether MoSwe1 is associated with vegetative growth in rice blast fungus, we observed the colony morphology of the Δ*Moswe1* mutant on complete medium (CM), minimal medium (MM), oatmeal agar medium (OMA), and V8 vegetable juice (V8) medium (Fig. 4A). Compared with the wild type 70-15, the colony size of Δ*Moswe1* was significantly reduced on CM, MM, OMA, and V8 medium after 9 days of growth. The Δ*Moswe1-C* recovered the defects of the Δ*Moswe1* mutant (Fig. 4B). To further understand whether MoSwe1 is also involved in appressorium development, the ratios of appressorium formation in 70-15, Δ*Moswe1* and the Δ*Moswe1-C* were determined at 4, 6, 8 and 24 hpi (Fig. 4C). At 4 hpi, barely 2.31% of the conidia in the Δ*Moswe1* mutant could form appressoria, compared with 34.67% in the wild type 70-15. At 24 hpi, the ratio of appressorium formation in Δ*Moswe1* increased to 47.79%. However, there were still significant differences compared with the proportions of appressorium formation in the 70-15 and Δ*Moswe1-C*, which exceeded 90% (Fig. 4D). The data showed that *MoSWE1* is indispensable for fungal vegetative growth and appressorium formation in *M. oryzae*.

Since conidia are the spearhead of the spread of *M. oryzae*, we also evaluated the role of MoSwe1 in conidiation and conidial morphology. Conidia from 70-15, Δ*Moswe1*, and the Δ*Moswe1-C* were collected at 8 days-post-incubation (dpi) after culture on CM plates. We found that the wild type 70-15 conidiation was approximately 500 times that of the Δ*Moswe1* mutant. This result indicated that the conidiation of the Δ*Moswe1* mutant decreased sharply (Fig. 4E). Interestingly, via CFW staining, we found that septum formation was significantly impaired in the Δ*Moswe1* mutant. Compared to more than 80% of conidia with two septa in wild type 70- 15, conidia in the Δ*Moswe1* mutant contained up to 3, 4, and 5 septa. This damage was fully recovered in the Δ*Moswe1-C* (Fig. 4F). As is shown in Fig. 4G, the percentage of conidia containing 4 cells, 5 cells, and 6 cells in the Δ*Moswe1* mutant made up almost 50% of the total conidia. In addition, the appressorial morphology in the Δ*Moswe1* mutant was changed. The appressoria of the Δ*Moswe1* mutant were smaller than those of 70-15, and the Δ*Moswe1-C*. The wild type 70-15 appressoria were ~ 10 μm, while the diameter of the Δ*Moswe1* mutant appressoria was only ~ 6 μm (Fig. 4H and I). Overall, the results clarified that *MoSWE1* plays essential roles in conidial septum formation and conidial and appressorial morphology in *M. oryzae*.

### MoSwe1 is involved in pathogenicity in *M. oryzae*

**Fig. 5:**
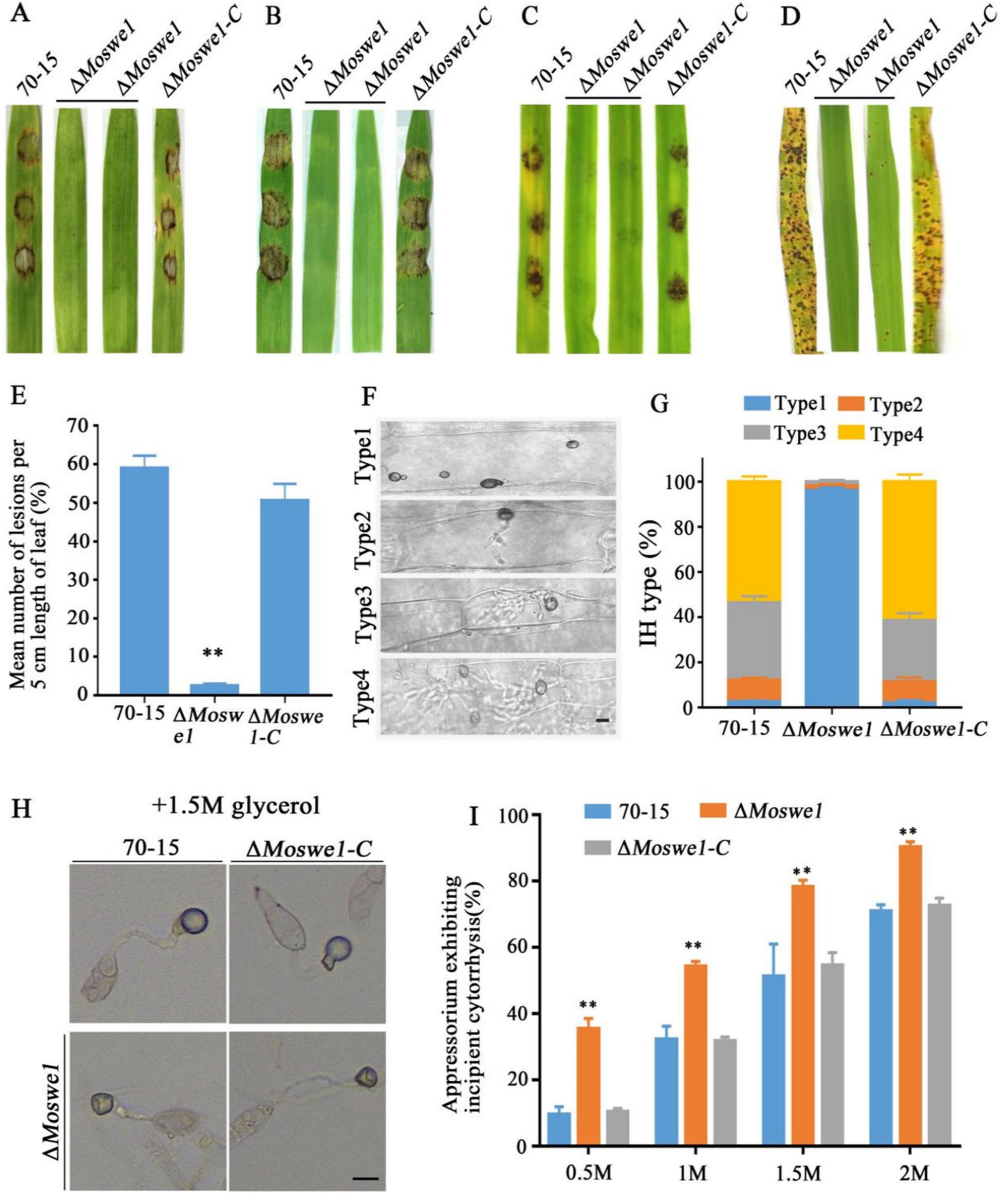
MoSwe1 contributes to virulence in *M. oryzae*. **A** Conidial suspensions (1 × 10^5^ conidia/ml) of the 70-15, Δ*Moswe1* and Δ*Moswe1-C* strains were dropped on two-week-old rice seedlings. **B** Conidial suspensions (1 × 10^5^ conidia/ml) of the 70-15, Δ*Moswe1*, and Δ*Moswe1-C* were dropped onto barley leaves in vitro. **C** Disease incidence on isolated barley leaves inoculated with mycelial plugs of the 70-15, the Δ*Moswe1* and Δ*Moswe1-C* strains. **D** Rice seedlings (2 weeks old) inoculated with conidial (5 × 10^4^ conidia/ml) suspensions of 70-15, Δ*Moswe1* and Δ*Moswe1-C*. Evaluation of disease spots on rice leaves was carried out at 7 dpi. **E** The lesion areas were counted and calculated using Adobe Photoshop CC. **F** and **G** Artificial division of the types of IH into four types. At least 100 IH of each strain were tallied. Scale bar, 10 μm. **H** and **I** Cell collapse experiments were performed with 0.5-2 M glycerol concentrations, and at least 100 appressoria were tallied for each concentration. Asterisks denote statistical significance (*P* < 0.01)

To study the role of *MoSWE1* in *M. oryzae*, we tested the virulence of the wild type 70-15 strain, Δ*Moswe1* mutant and the Δ*Moswe1-C* on detached susceptible rice seedling leaves and (*Oryza sativa* cv. CO-39) and barley leaves (*Hordeum vulgare* cv. ZJ-8). The Δ*Moswe1* mutant showed fewer lesions than the wild type 70- 15 and the Δ*Moswe1-C* (Fig. 5A and B). Furthermore, we found that the disease spots on barley leaves inoculated with the Δ*Moswe1* mutant did not extend (Fig. 5C), indicating that MoSwe1 affected the invasive growth of rice blast fungus. We found that the Δ*Moswe1* mutant almost did not infect rice leaves (Fig. 5D), and the disease spots caused by the Δ*Moswe1* mutant on rice leaves were virtually ignored (Fig. 5E). In addition, microscopic observations confirmed that almost all types of invasive hyphae (IH) in Δ*Moswe1* were type 1 (type 1, no IH; type 2, 1–2 IH; type 3, at least 3 short IH; type 4, abundant IH) at 48 hpi, indicating that the Δ*Moswe1* appressoria barely penetrated plants, whereas more than 90% of the IH in 70-15 and the Δ*Moswe1-C* were types 3 and 4 (Fig. 5F and G). Moreover, it was found that the appressorial collapse rate of the Δ*Moswe1* mutant was dramatically higher than those of the 70-15 and Δ*Moswe1-C* (Fig. 5H and I). The appressorial collapse rate of the Δ*Moswe1* mutant was 78.15% compared with collapse rate of 51.14% in the wild-type 70-15 when the concentration of glycerol up to 1.5 M. Based on these experimental results, we concluded that MoSwe1 take on crucial roles during *M. oryzae* infection.

### MoSwe1 interacts with MoAtg17 and MoAtg18 and regulates autophagy

**Fig. 6:**
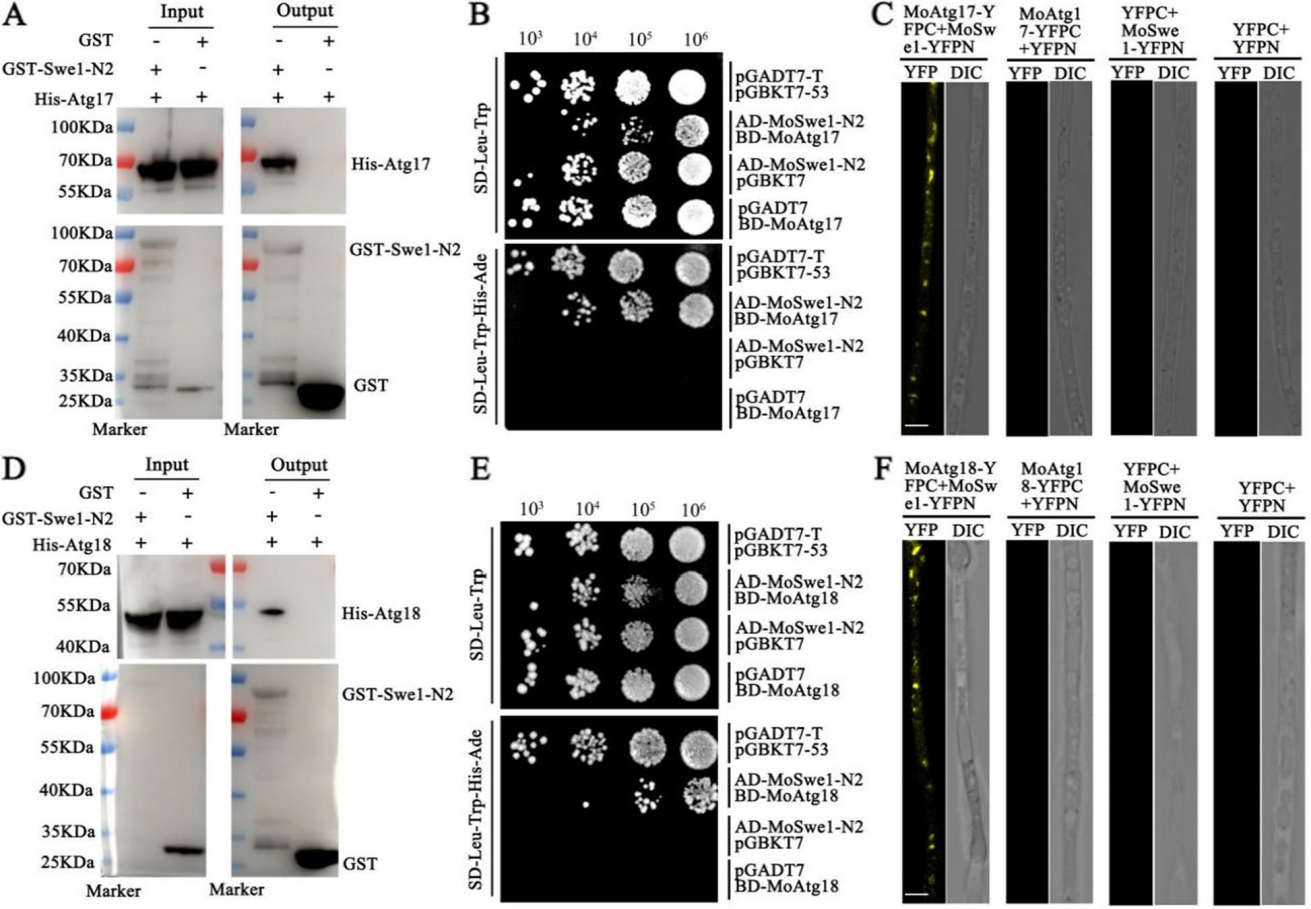
MoSwe1 interacts with MoAtg17 and MoAtg18. **A** and **D** Pull-down assays. GST magnetic beads were used to pull down MoSwe1-N2-GST protein, which was enriched on the magnetic beads, and MoAtg17-His or MoAtg18-His was added and eluted. Anti-His and anti-GST antibodies in elution buffer detected the presence of MoAtg17-His or MoAtg18-His. **B** and **E** Yeast two-hybrid experiment to test the interaction between MoSwe1 and MoAtg17 and MoAtg18. **C** and **F** BiFC experiment to test the interaction between MoSwe1 and MoAtg17 and MoAtg18. Scale bar, 5 μm

To further elucidate the regulatory mechanism of MoSwe1, we conducted yeast two-hybrid (Y2H) assays to identify potential interactions. Concurrently, Y2H assays with a series of truncated MoSwe1 constructs revealed that the protein kinase C-terminal domain is essential for MoSwe1-MoAtg17 and MoSwe1-MoAtg18 interactions (Fig. 6B and E). Since the C-terminal domain of MoSwe1 is necessary for the interaction, we generated a MoSwe1-N2-GFP complemented construct (a truncated MoSwe1 containing a C-terminal domain [726-1110 aa] fused with GFP). We transformed it into Δ*Moswe1* to investigate the function of the protein kinase C-terminus. The strain Δ*Moswe1-N2*-GFP displayed reduced mycelial growth on CM, although the growth defect was not as severe as that of Δ*Moswe1* (Fig. S4A). In addition, the pathogenicity of the Δ*Moswe1-N2*-GFP strain did not fully recover and was only slightly more potent than that of Δ*Moswe1* (Fig. S4B). Next, we carried out a pull-down experiment, and the results further verified the interaction between MoSwe1 and MoAtg17, and MoAtg18 (Fig. 6A and D). To find out MoSwe1 with MoAtg17 as well as the relationship between MoAtg18, we used a BiFC experiment to verify the interaction of MoSwe1 with MoAtg17 (Fig. 6C) and MoAtg18 (Fig. 6F). Taken together, these results convincingly demonstrated that MoSwe1 physically interacts with MoAtg17 and MoAtg18 in *M. oryzae*.

**Fig. 7:**
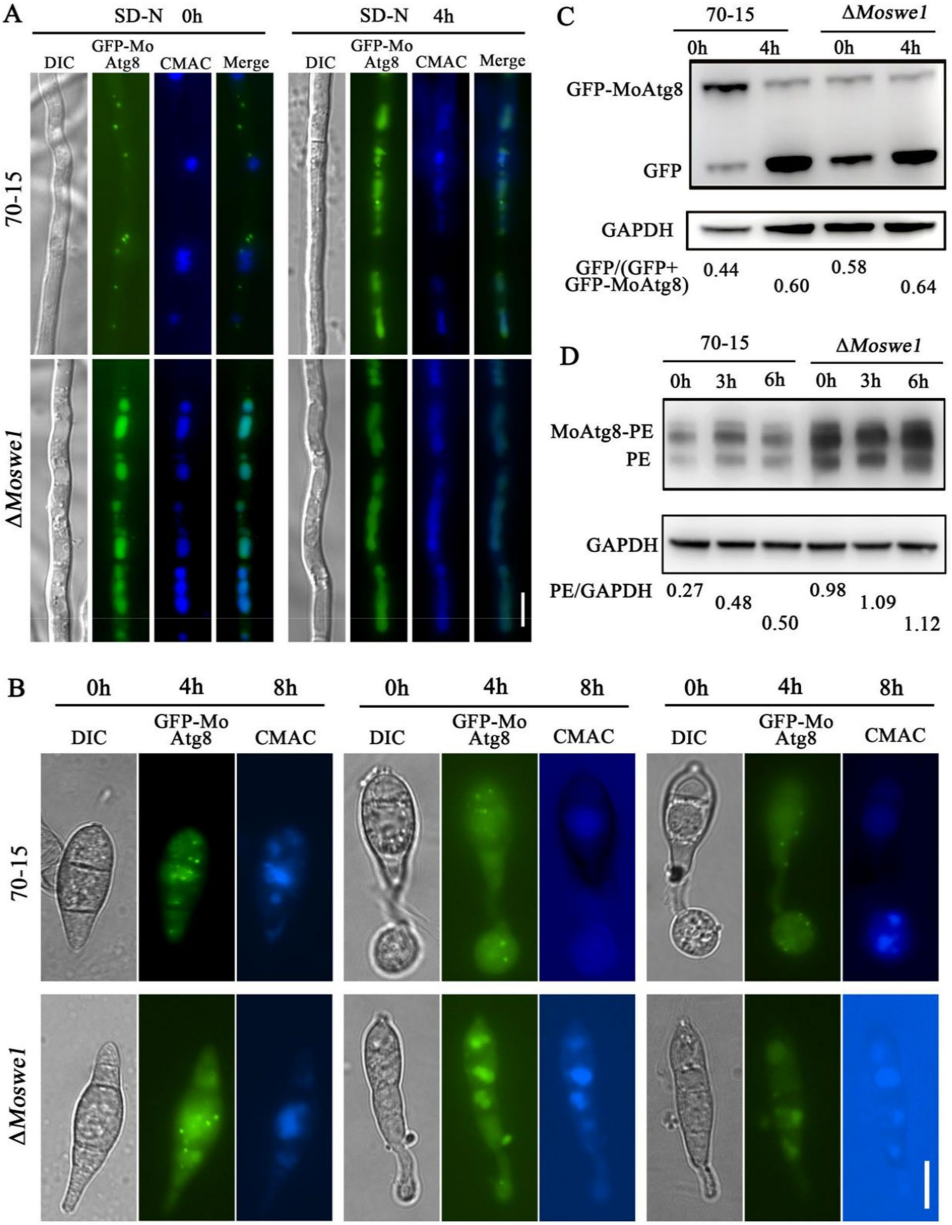
MoSwe1 negatively regulates autophagy in *M. oryzae*. **A** The localization of GFP-MoAtg8 in 70-15, the Δ*Moswe1* mutant. The hyphae of 70-15 and the Δ*Moswe1* mutant were grown in liquid CM for 2 days and then transferred to SD-N medium for 4 h. Hyphae were stained with 7-amino-4-chloromethylcoumarin (CMAC). Scale bar, 10 μm. **B** Conidial autophagy in 70-15 and Δ*Moswe1* mutant. Conidia were stained with CMAC. **C** Immunoblot analysis of the breakdown of GFP-MoAtg8 of 70-15 and Δ*Moswe1* mutant under nitrogen starvation conditions for 4 h. **D** Observation of MoAtg8/MoAtg8-PE turnover in 70-15 and Δ*Moswe1* mutant. Scale bar, 10 μm

Studies have shown that the infection-associated nuclear degradation in *M. oryzae*, which is required to cause rice blast disease, occurs by non-selective autophagy [22]. To examine whether MoSwe1 is necessary for the autophagy process, we evaluated autophagic activity by a survey of GFP-MoAtg8 processing. When cultured in CM medium (0 h), GFP-MoAtg8 fluorescence spots in the wild type 70-15 were mainly located in the cytoplasm near the vacuole. In contrast, GFP-MoAtg8 fluorescence spots in the Δ*Moswe1* mutant were primarily located in the vacuole. After 4 h induction with nitrogen starvation medium (SD-N) medium, a small fraction of GFP-MoAtg8 fluorescence spots were found in the cytoplasm. They did not degrade into vacuoles in the wild type 70-15. In the Δ*Moswe1* mutant, GFP-MoAtg8 fluorescence spots were located in the vacuole, and almost no dots were in the cytoplasm (Fig. 7A).

We collected conidia of wild type 70-15 and the Δ*Moswe1* mutant to observe the autophagy processes associated with infection during appressorium development. GFP-MoAtg8 was mainly located in the cytoplasm from 0 to 4 h, and almost no fluorescence spots were observed in the cytoplasm at 8 h in the wild type 70-15. In the Δ*Moswe1* mutant, there were very few fluorescent spots in the cytoplasm from 0 to 12 h (Fig. 7B). We then examined the autophagic flux in wild type 70-15 and the Δ*Moswe1* mutant. In the wild type 70-15, the degradation of GFP-MoAtg8 was inhibited under the condition of nutrition, but the content of free GFP in the Δ*Moswe1* mutant was higher than that in the wild type 70-15. The results indicated a significant increase in autophagy flow in the Δ*Moswe1* mutant. After 4 h of starvation induction with SD-N medium, the bands of GFP-MoAtg8 in the Δ*Moswe1* mutant almost disappeared (Fig. 7C), indicating that the autophagy process was accelerated and the autophagy was maintained at a high level in the Δ*Moswe1* mutant. We also investigated the turnover of MoAtg8/MoAtg8-PE, and we found that the lipidation level of MoAtg8 in the Δ*Moswe1* mutant was significantly higher than that of the wild type 70-15 (Fig. 7D). This suggests that MoSwe1 negatively regulates autophagy.

### MoSwe1 interacts with MoMps1 and affects the phosphorylation of MoMps1

**Fig. 8:**
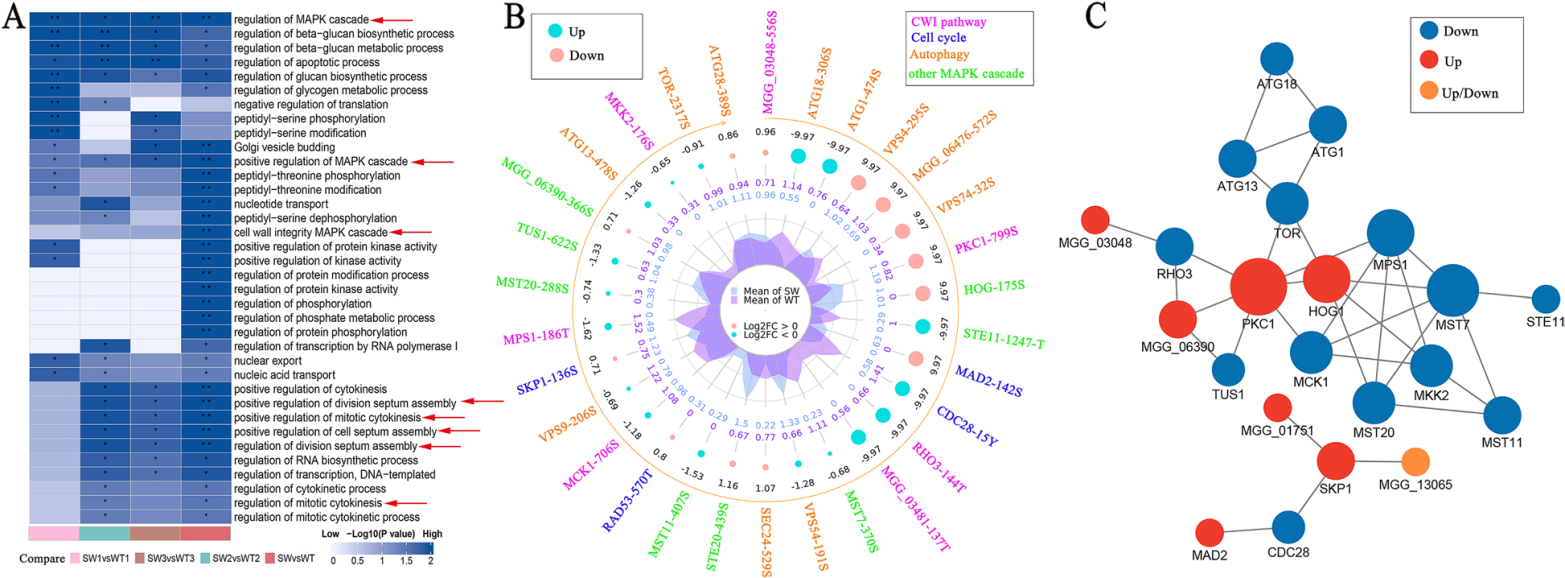
Phosphorylation difference between 70-15 and Δ*Moswe1* mutant was analyzed by phosphoproteome. **A **After GO classification, KEGG pathway, protein domain, Reactome, and WikiPathways enrichment, cluster analysis was conducted to find differences and similarities in the function of differentially expressed modified proteins between the comparison groups. There were three replicates of 70-15 and the Δ*Moswe1* mutant. SW represents Δ*Moswe1* mutant, and WT represents wild type 70-15. **B** We used radar maps to show the proteins that significantly expressed the autophagy, CWI, and cell cycle pathways among the differentially expressed proteins. From the outside to the inside, the first circle represents multiple differential proteins; the second circle orange arrow represents the sequence of differential proteins from small to large according to the *P* value; the third circle represents the ratio of differential expression change in the comparison group of Log2 conversion; pink represents up-regulation, blue represents down-regulation; the more significant the dot, the larger the multiple of difference. The fourth circle represents the average quantitative value of the two groups. **C** The differentially expressed proteins in the CWI pathway, autophagy pathway, and cell cycle were screened out from the significantly differentially expressed proteins. After comparing with the STRING protein interaction network database, the differentially modified protein interaction was extracted according to the confidence score > 0.7 (high confidence). Circles represent differentially modified proteins, blue are down-regulated modified proteins, red are up-regulated modified proteins, yellow is modified proteins that contain both up and down-modified sites, and the size of the circles indicate the number of proteins with which they interact

We have demonstrated that MoSwe1 affects MoCdc28 phosphorylation as a kinase in *M. oryzae*. To explore whether MoSwe1 affects phosphorylation levels in other pathways, we carried out quantitative phosphoproteomic analyses. Phosphoproteins were purified from Δ*Moswe1* and 70-15 by liquid chromatography with tandem mass spectrometry (LC-MS/MS). The data revealed MoSwe1-dependent phosphorylation of proteins associated with the cell cycle, autophagy, and MAPK cascade (Fig. 8A).

As shown in the radar map of differentially modified sites (Fig. 8B), proteins with significantly different relative expression levels at differentially modified sites were divided into 4 categories. Among the 7 related proteins in the CWI pathway, *PKC1* and MGG_03048 were significantly up-regulated, while *RHO3*, MGG_03481, *MCK1*, *MPS1*, and *MKK2* were significantly down-regulated. All proteins related to the cell circulation pathway were significantly up-regulated except *CDC28*. In autophagy pathway, *ATG28*, *VPS4*, *VPS74*, MGG_06476, *SEC24* were significantly up-regulated, *ATG1*, *ATG13*, *ATG18*, *VPS9*, *TOR* and *VPS54* were significantly down-regulated. Through protein interaction network analysis, it was found that autophagy pathway-related proteins and MAPK pathway-related proteins had an interaction relationship. Still, the two pathway-related proteins had no interaction relationship with cell cycle pathway-related proteins (Fig. 8C).

**Fig. 9:**
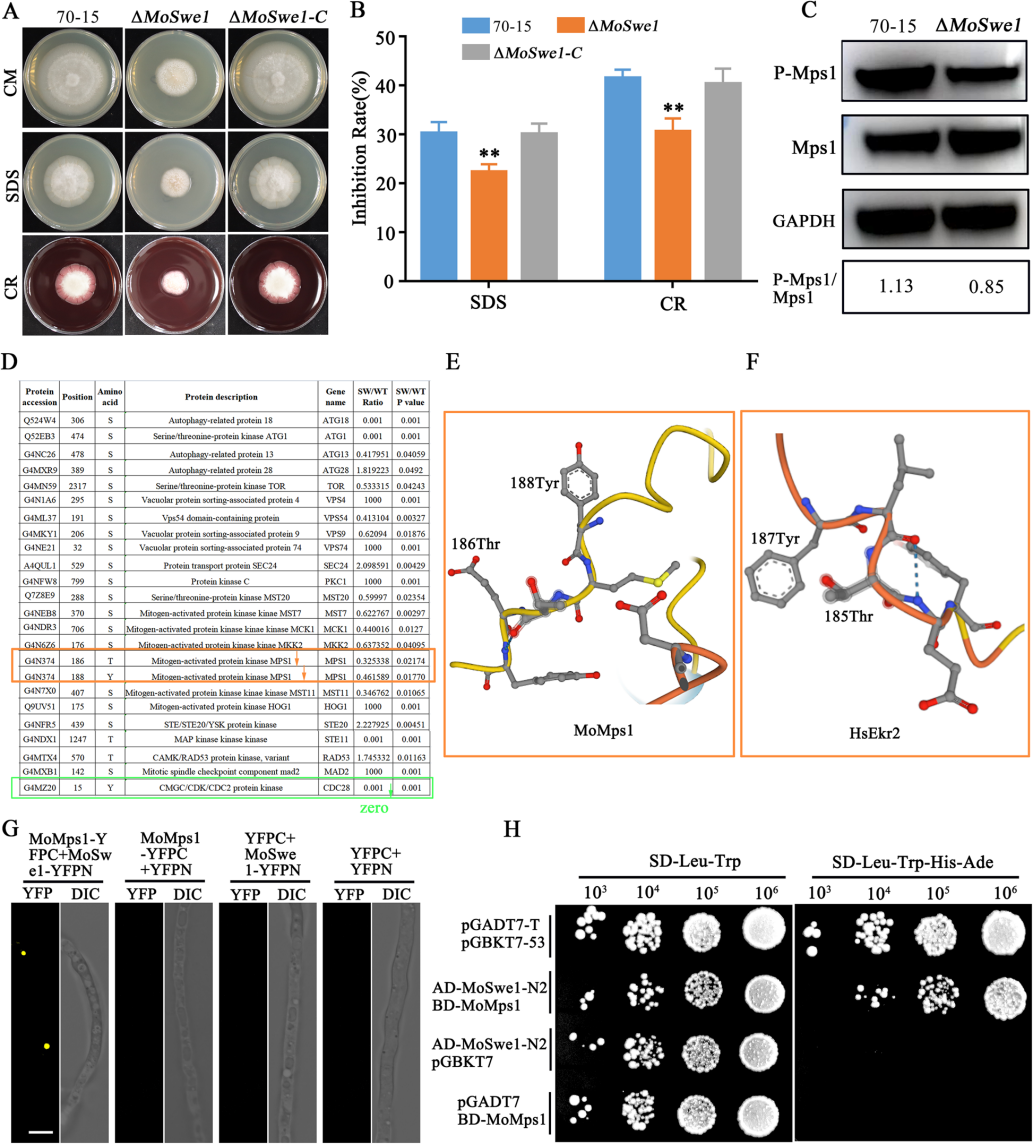
MoSwe1 affects the CWI pathway by regulating MoMps1 phosphorylation. **A** 70-15, the Δ*Moswe1* mutant, and Δ*Moswe1*-C on CM with 0.005% SDS, 400 µg/ml Congo Red (CR) were cultured for 8 days, and pictures were taken. **B** The relative inhibition rates of 70-15, Δ*Moswe1* mutant, and Δ*Moswe1*-C were calculated in triplicate. Asterisks are used to indicate statistically significant differences (**P < 0.01). **C** The phosphorylation level of Mps1 in 70-15 and the Δ*Moswe1* mutant was assayed. **D** Phosphoproteomic data showed that MoMps1 phosphorylation levels at 186T and 188Y were significantly down-regulated. **E** and **F** AlphaFold predict Mps1 of *M. oryzae* and human homologous Mps1 and intercept amplification near the phosphorylation site. **G** BiFC experiment verifies the interaction between MoMps1 and MoSwe1. Scale bar, 5 μm. **H** The interaction between MoMps1 and MoSwe1 was verified by a yeast two-hybrid experiment

Based on the phosphoproteomic data, we speculated that MoSwe1 affected the MAPK pathway. Sensitivity tests showed that the Δ*Moswe1* mutant grew faster in the medium containing 0.005% sodium dodecyl sulfate (SDS) and 400 ug/ml Congo red (CR), and the Δ*Moswe1* mutant showed higher resistance to SDS and CR (Fig. 9A and B). We then used P-p44/42 antibody and found that MoMps1 phosphorylation levels were significantly reduced in the Δ*Moswe1* mutant. According to the phosphoproteomic data, the phosphorylation levels of MoMps1 thr186 and tyr188 sites were down-regulated considerably (Fig. 9D). MoMps1 in *M. oryzae* was homologous to *Homo sapiens* Erk2. The thr186 and tyr188 protein sites of MoMps1 (Fig. 9E) were consistent with the thr185 and tyr187 protein sites of Erk2 (Fig. 9F), respectively. The anti-phospho-p44/42 MAPK antibody (P-p44/42) detects p44 and p42 MAP kinases (Erk1 and Erk2) at endogenous levels after thr202 and tyr204 (Thr185 and Tyr187 of Erk2) double phosphorylation and after thr202 monophosphorylation [12, 58]. The interaction between MoSwe1 and MoMps1 was proved by the BiFC experiment (Fig. 9G) and yeast two-hybrid (Fig. 9H). In conclusion, we suggest that MoSwe1 interacts with MoMps1 to influence the infection and pathogenicity of *M. oryzae* by inhibiting the phosphorylation of MoMps1 185T and 187Y sites.

### MoSwe1 affects septin ring assembly at the appressorium pore in *M. oryzae*

The cell cycle regulates septin ring formation, and the septin ring affects appressorial penetration [61]. In *M. oryzae*, septin ring assembly affords a scaffold for the annular F-actin network at the appressorium pore [11]. Therefore, studying the septin ring assembly can clarify the relationship between cell cycle progression and appressorial penetration. We speculated that MoSwe1 affects septin dynamics at the appressorium pore, which results in the penetration defect of the Δ*Moswe1* mutant in the rice blast fungus. We expressed MoSep5-GFP in the wild type 70-15 and the Δ*Moswe1* mutant to investigate this hypothesis. The wild type 70-15 formed a bright ring at the appressorium pore. In contrast, the Δ*Moswe1* mutant formed an aberrant ring instead of the typical central septin ring in the appressorium pore (Fig. S4C and D). The results showed that the septin ring assembly in the appressorium pore was disrupted in the Δ*Moswe1* mutant.

### Glycogen and lipid utilization and degradation are attenuated in the Δ*Moswe1* mutant

 In *M. oryzae*, efficient utilization and transfer of glycogen and lipids contained in conidia are necessary for the appressoria to penetrate plant leaves [2, 67]. In the wild type 70-15, a large amount of glycogen accumulates in the ungerminated conidia but is immediately degraded during conidial germination. During appressorium development, as the appressorium begins melanism, the abundant glycogen in it begins to degrade [67]. Changes in glycogen position during appressorium formation were observed by iodine staining. Large amounts of glycogen were observed in the conidia, germ tubes, and incipient appressoria of wild type 70-15 and the Δ*Moswe1* mutant from 0 to 4 h during conidial germination (Fig. S5A and B). However, the redeployment of glycogen was remarkably retarded in the Δ*Moswe1* mutant at 16 hpi. While 80.13% of the glycogen in conidia of wild type 70-15 was transferred into the appressoria, 66.7% of the glycogen in the Δ*Moswe1* mutant was not transferred to appressoria (Fig. S5C and D). At 24 hpi, only 41.73% of the glycogen in Δ*Moswe1* appressoria had been degraded and utilized, while 95.17% of that in wild type 70-15 appressoria had been degraded and used (Fig. S5C and E). As shown in Fig. S5F-H, consistent glycogen and lipid body utilization and degradation patterns were observed in the Δ*Moswe1* mutant. The results indicated that the utilization and degradation of glycogen and lipid bodies are attenuated in the Δ*Moswe1* mutant.

### Glycogen metabolism, fatty acid metabolism, and autophagy pathways were disturbed in the Δ*Moswe1* mutant

Considering that MoSwe1 functions in glycogen metabolism and lipid metabolism progression and influences autophagy, the metabolomic and transcriptomic profiles of Δ*Moswe1* and 70-15 were analyzed and compared. Kyoto Encyclopedia of Genes and Genomes (KEGG) enrichment analysis indicated that galactose metabolism, fructose, and mannose metabolism, biosynthesis of unsaturated fatty acids, and fatty acid biosynthesis were the most highly enriched pathways (Fig. S6A). A cluster map of the differential metabolites, including fatty acids (alpha-linolenic acid, linoleic acid, tetracosanoic acid, stearic acid, palmitic acid, myristic acid, pentadecanoic acid, 11(Z),14(Z),17(Z)-eicosatrienoic acid) and glycogen (sucrose, maltotriose, D-quinovose, alpha-D-glucose, D-mannose), showed higher levels in the Δ*Moswe1* mutant (Fig. S6B). Transcriptome data analysis again indicated that MoSwe1 regulates many metabolic pathways by regulating the expression of the other genes involved in glycerolipid metabolism, mannose type O-glycan biosynthesis, galactose metabolism, glycerophospholipid metabolism, amino sugar and nucleotide sugar metabolism, pentose and glucoronate interconversions, fructose and mannose metabolism, starch and sucrose metabolism, and autophagy (Fig. S6C).

## Discussion

The cell cycle is critical for differentiation in eukaryotic cells and must effectively coordinate with cell division to form specific tissues [10, 29, 66]. The Cell wall integrity (CWI) signaling pathway is necessary for maintaining cell morphology and acting as a barrier against extracellular stress [42]. At the same time, The autophagy pathway degrades waste organs and other materials so that cells can survive better [80]. The association between the autophagy pathway and the CWI pathway was established by phosphorylation of the CWI pathway protein MoMkk1 by the autophagy protein MoAtg1 in *M. oryzae* [79]. The CWI pathway protein MoMkk1 was phosphorylated by Mitotic Exit Network (MEN) protein MoSep1 to link the cell cycle pathway to the CWI pathway in *M. oryzae* [13]. However, the relationship between autophagy, CWI, and cell cycle pathways remains unclear. In our study, we found that a cell cycle kinase, MoSwe1, phosphorylates the CWI pathway MoMps1, linking the CWI pathway to the cell cycle pathway. In addition, MoSwe1 interacts with MoAtg17 and MoAtg18 to affect autophagy pathways, linking cell cycle pathways to autophagy pathways. phosphoproteomic data also showed significant changes in the phosphorylation levels of autophagy pathway associated proteins, CWI associated proteins, and mitosis associated proteins in the Δ*Moswe1* mutant. MoSwe1 connects cell cycle pathway, CWI pathway, and autophagy pathway in series. More importantly, the absence of *MoSWE1* seriously affected the infection and pathogenicity of *M. oryzae*.

Swe1-related kinase function through a highly conserved mechanism that controls the timing of entry into mitosis and monitors cell size or growth [27]. In *S. cerevisiae*, *∆swe1* cells undergo premature entry into mitosis before sufficient growth of the daughter bud has occurred, producing abnormally small cells [20]. Swe1 regulates the G2/M transition in *S. cerevisiae* [7]. Studies have shown that the *Swe1*-lacking mutant does not enter mitosis in advance in budding yeast [7, 63]. Studies in fission yeast and *Xenopus* have shown that mutants enter mitosis prematurely [51, 71]. Compared with the results obtained thus far for model organisms in yeast and animals, our finding here revealed that the *M. oryzae* homologs of *SWE1*, namely, *MoSWE1*, play a primary role in phase progression from G1 to S but not the other phases. This novel discovery is confirmed by the following results. HU treatment triggers the S phase checkpoint and arrests cells in S phase [34]. In our study, the Δ*Moswe1* mutant and wild-type strain 70-15 showed no difference when treated with HU. Similarly, *Swe1* mutants in yeast did not show obvious HU sensitivity [34]. We speculated that MoSwe1 does not directly act on the S phase to regulate DNA synthesis. In fission yeast, a certain proportion of Δ*swe1* mutant cells were multinucleated, with some having as many as eight nuclei [20]. The number of conidial septa and nuclei in each conidial cell was increased in the Δ*Moswe1* mutant. The cells generally exhibited two nuclei; some conidia had up to eight nuclei. Using chromosomal tags, we also examined whether the cells were in the G1 or S/G2 phase of the cell cycle. We found that all cells of the conidia in the Δ*Moswe1* mutant entered the S phase, not just the apical cells, and the appressoria also entered the S phase again to replicate, forming four GFP spots. This finding suggested that knocking out *MoSWE1* leads to premature entry into the S phase, and excessive mitosis leads to developmental abnormalities. MoSwe1 is similar to the switch of the mitotic process. After *MoSWE1* knockout, the mitotic process of the rice blast fungus was uncontrolled, and the fungus continued to divide. Therefore, we concluded that MoSwe1 regulate the G1/S phase transition in cell cycle progression of *M. oryzae*.

Swe1 plays a crucial role in morphogenesis checkpoints that delay cell cycle progression in response to defects in bud formation [39, 47]. In *M. oryzae*, the S phase is essential for the differentiation of the initial appressorium, while the M phase is necessary to develop the functional appressoria that form the penetration peg [61]. We found that the conidial morphology of the Δ*Moswe1* mutant was abnormal; most of the conidia were longer, and the number of conidial septa was increased, with three-cell conidia in wild type 70-15 and up to 4/5/6 cells in one conidium of the Δ*Moswe1* mutant. In addition, the appressoria of the Δ*Moswe1* mutant were smaller in size than the appressoria of 70-15. MoSwe1 might play an essential role in monitoring cytoskeletal organization in *M. oryzae*.

Swe1 is essential for mitosis, but other specific functions of Swe1 in pathogenic plant fungi are still unclear. The absence of any CWI MAPK cascade component has resulted in reduced pathogenicity of *M. oryzae*. The CWI pathway is necessary for the hyphae morphogenesis and infection process of *M. oryzae* [78]. Cell cycle-related proteins are also essential for maintaining appressorium development and pathogenicity in *M. oryzae* [17, 62]. Pkc1 (CWI pathway-associated protein) negatively regulates the cell cycle G2/M transcriptional program [18]. CWI pathway is activated during the budding process and inactivated in the G2/M transition [30, 43]. Here, we discovered that the Δ*Moswe1* mutant was more resistant to cell wall stress. MoSwe1 interacted with MoMps1. MoSwe1 affected MoMps1 phosphorylation by phosphorylating MoMps1 at the thr185 site and tyr187 site.

Autophagy is a highly conserved catabolic pathway that uses the autophagosome to enclose cellular materials and fuse them with lysosomes to degrade them, then release and recycle them to support cell metabolism [33]. Studies have shown that autophagy activity is reduced during mitosis in human cells. Cyclin-dependent kinase inhibitors (CDKI) and retinoblastoma protein (Rb)/E2 factor (E2F) regulate autophagy activity [4, 43]. Here, we found that MoSwe1 interacts with MoAtg17 and MoAtg18. The phosphorylation levels of MoAtg1 and MoAtg18 were significantly down-regulated in the Δ*Moswe1* mutant. In the Δ*Moswe1* mutant, the autophagy process was markedly accelerated since the content of MoAtg8-PE was increased considerably. Our results suggested that MoSwe1 negatively regulates autophagy by promoting the phosphorylation levels of MoAtg17 and MoAtg18.

In *M. oryzae*, the assembly of septin and the establishment of the F-actin network are necessary for the rice blast fungus to rupture the leaf cuticle plant [11]. In the Δ*Moswe1* mutant, the frequency of the septin ring was lower than that in the wild type 70 − 15. The results indicated that the attenuated assembly of septin in the Δ*Moswe1* mutant decreased the cortical rigidity at the appressorium pore enough that the fungus could not penetrate the host cuticle. In *M. oryzae*, from conidium to appressorium formation, mitosis is always accompanied by programmed cell death of the conidia [68]. Lipid and glycogen stores in the conidia are mobilized and transported to the appressorium during appressorium development in *M. oryzae*. Previous studies have indicated lipid metabolism and the glyoxylate cycle are necessary for appressorium formation rather than sugar metabolism [5, 6, 55, 57, 72, 73]. Richard A. Wilson proved that *TKL1*, encoding transketolase, is required for a metabolic checkpoint to control the cell cycle and hyphal growth in *M. oryza*e [14]. *ABL1* acts as a glucose signal component to regulate cell cycle tuning and mediate the terminal appressorium of the cell differentiation in *M. oryzae* [44]. In the Δ*Moswe1* mutant, the mobilization of glycogen and lipids was notably retarded. At 24 hpi, undegraded glycogen and lipids remained in the conidia in the Δ*Moswe1* mutant. Metabolomics and transcriptome data showed that glycogen and fatty acid metabolism in the Δ*Moswe1* mutant significantly differed from those in the wild type 70-15. These data indicated that *MoSWE1* is related to glycogen and fatty acid metabolism. However, how MoSwe1 specifically responds to sugar metabolism, fatty acid, or energy metabolism to regulate the cell cycle remains to be further studied.

The Nicholas J. Talbot laboratory has certified that rice blast fungus infection is controlled by two unrelated S-phase checkpoints that operate in two consecutive cell cycles in the blast fungus [53]. Only when the second round of mitosis proceeds normally can the penetration peg elongate and breach the cuticle of the leaves [52]. In our study, the appressorial collapse rate of the Δ*Moswe1* mutant was strikingly higher than that of wild type 70-15, indicating that the turgor pressure in the appressorium was affected in the Δ*Moswe1* mutant, which further affected plant infection. Therefore, nearly no invasive hyphae were formed after 48 h of induction on the leaves treated with the Δ*Moswe1* mutant, and thus, the Δ*Moswe1* mutant is nearly nonpathogenic. In previous research, Osés-Ruiz et al. speculated that in the process of plant penetration mediated by appressoria in *M. oryzae*, there might be a close relationship between turgor generation, cytoskeletal reorganization, and cell cycle progression by the MoSwe1 kinase [52]. These data indicated that MoSwe1 regulates the turgor pressure, which is dependent on the second round of the cell cycle.

**Fig. 10:**
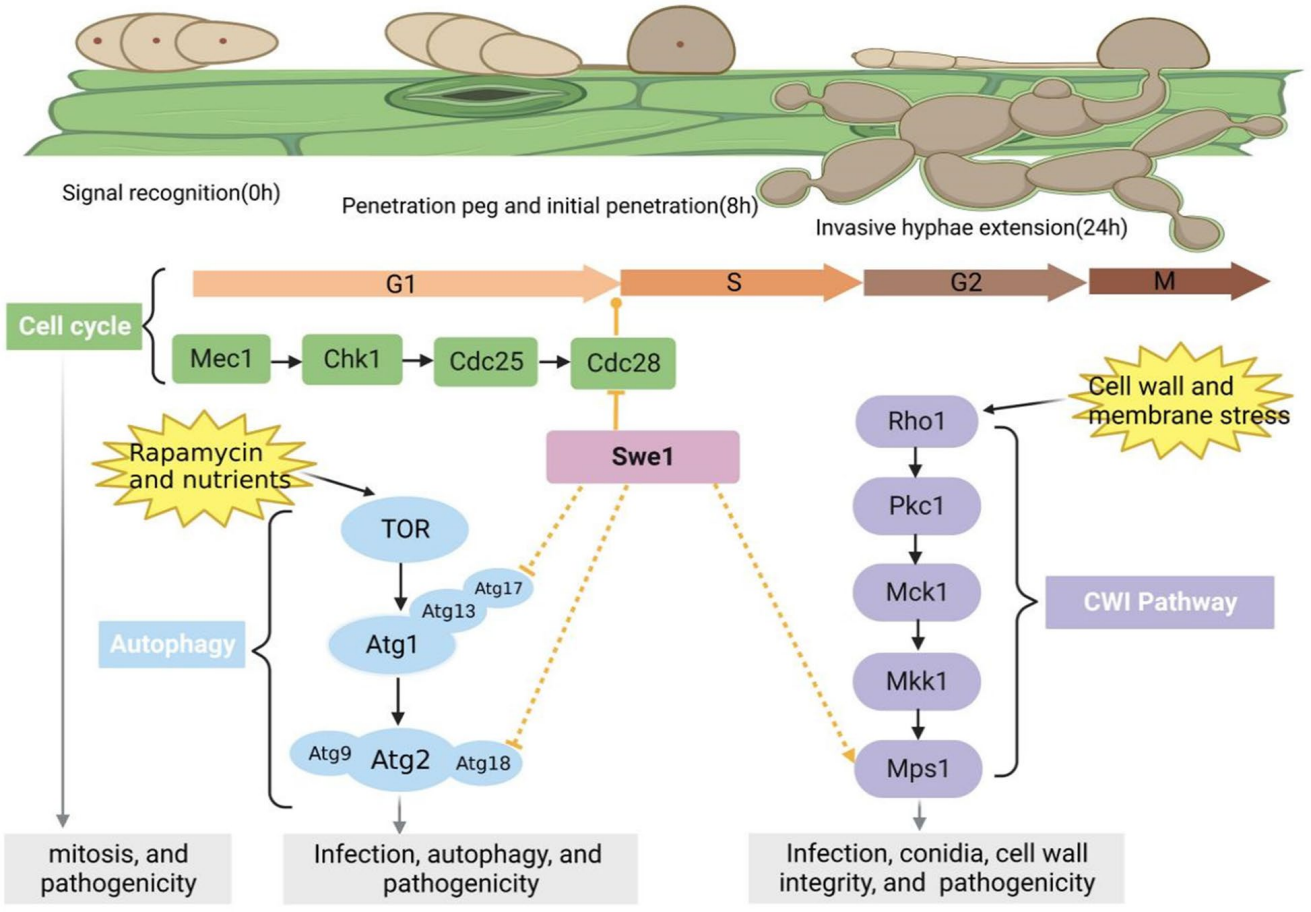
A proposed model for the MoSwe1 function. In the process of *M. oryzae* infection, it faces various stresses from the external environment and rice. MoSwe1, as a mitotic protein kinase, is involved in regulating the G1 to S phase of *M. oryzae* mitosis but not other phases. Swe1 negatively regulates autophagy by interacting with MoAtg17 and MoAtg18. MoSwe1 affects MoMps1 phosphorylation and thus regulates the conidiation and CWI pathway of *M. oryzae*. MoSwe1 connects the cell cycle, CWI pathway, and autophagy to coordinate the regulation of the pathogenicity of *M. oryzae*

In conclusion, the study of MoSwe1 regulating MoMps1 phosphorylation reveals a new mechanism by which the cell cycle and CWI pathway coordinate to influence the pathogenesis of *M. oryzae*. Meanwhile, the interaction of MoSwe1 with MoAtg17 and MoAtg18 revealed the coordination of the cell cycle and autophagy to regulate the pathogenesis of *M. oryzae* (Fig. 10). Such a conclusion implies that the function of the cell cycle in response to the CWI pathway and autophagy plays an integral part in the growth and pathogenicity of the rice blast fungus. Given such an important role, further study of cell cycle-CWI-autophagy crosstalk is imperative.

## Materials and methods

### Strains and cultural conditions

The wild-type strain of *M. oryzae* applied for this study was 70-15. Strains were grown on CM in a constant temperature (25°C) incubator alternating between 16 h of light and 8 h of darkness [54] unless indicated otherwise.

### *MoSWE1* gene disruption and complementation

We constructed *MoSWE1* knockout vectors according to a previously described protocol [8, 40]. Primers (Table S1) were used to amplify upstream (~ 1000 bp) and downstream (~ 1000 bp) segments from the wild type 70 − 15. Special primers were applied to magnify the *BAR* (glufosinate acyltransferase) sequence from pCB1003 [36]. The pKO1B vector was cut open with a restriction endonuclease, and then the three recovered purified fragments (upstream, downstream, *BAR*) were joined to the cut vector by fusion enzyme (Vazyme, China) [8]. The correctly constructed knockout vector verified by PCR was used to transform 70 − 15 via *Agrobacterium tumefaciens*-mediated transformation (ATMT). The number of copies of the *BAR* gene in the mutant was detected by the fluorescent quantitative PCR (qPCR) method to determine whether the knockout mutant was a single-copy mutant. A single copy of the tubulin gene was used as a positive control. The data were computed according to a previous method [40]. To produce a Δ*Moswe1-C*, a *MoSWE1* gene containing an approximately 2000 bp promoter sequence was amplified and cloned into pKD5 [8].

### Mutant phenotyping experiments

For *M. oryzae* vegetative growth, small blocks of strains were cultured on CM medium plates for 8 days and then measured and analyzed. We used a previously described CFW staining method to assess conidial morphology to observe conidia separation [19]. The wild type 70-15 and the Δ*Moswe1* mutant were cultured on solid CM medium for 6 days at 25°C. The edge of the colony was selected to obtain a fungus cake by using a hole punch, and the cake was placed on a 9 cm petri dish and cultured for 11 days. Conidia were washed with distilled water and collected by centrifugation. The number of conidia was counted under the microscope with a hemocytometer. To assess conidial germination and appressorium formation of *M. oryzae*, 20 µl of conidial suspension (5 × 10^4^ conidia/ml) was inoculated onto a hydrophobic plastic surface and incubated in a humid box at 25°C in the dark. The assays were repeated independently three times, with three replicates each time. For the virulence test of *M. oryzae*, 20 µl of conidial suspensions (1 × 10^5^ conidia/ml) was dripped on susceptible rice seedling leaves and one-week-old barley leaves. In addition, isolated barley leaves were inoculated with mycelial plugs for 4 days. 1 × 10^5^ conidia/ml conidial suspensions were dropped on detached barley leaves at 25°C for the plant infection assays. The infection of barley leaves after 48 h of induction was surveyed with a Nikon microscope.

### Fluorescence observation

To study nuclear division, the nuclear localization signal with red fluorescence (NLS-mCherry) was diverted to *M. oryzae* wild type 70-15, the Δ*Moswe1* mutant, and the Δ*Moswe1-C*. The nuclear distribution in conidia and during appressorium development was observed by laser confocal microscopy (LSM880).

### Cytorrhysis analysis and HU assays

Mature appressoria were placed in glycerol at different concentrations, and at least 100 appressoria were tested in *M. oryzae* at each concentration. For HU treatment, 200 mM HU was added 2 h after the induction of appressoria, and appressorium formation was observed 24 h after induction in *M. oryzae* [18].

### Strains expressing GFP fusion protein for chromosome tagging

The chromosomal tag was obtained by simultaneously transforming the plasmid encoding the GFP-Lac repressor protein with the nuclear localization signal (pBITEFGFPLacIN) into the *M. oryzae* wild type 70-15, the Δ*Moswe1* mutant together with the plasmid encoding a collection of 256 lac operator repeats (pCTLacOS) [16]. Protoplasts were transformed as described previously [36].

### Quantitative real-time PCR (qPCR) methods

Wild type 70-15 and Δ*Moswe1* mutant strains were shaken and cultured in CM liquid medium for 48 h, the mycelia were collected, and the RNA was extracted after grinding with liquid nitrogen and reverse transcribed into cDNA. cDNA samples can be directly used in qPCR. We used a real-time PCR instrument (Eppendorf, Germany) to perform quantitative real-time PCR (qPCR) with ChamQ SYBR qPCR Master Mix (Vazyme, China) and measured gene expression levels according to the manufacturer’s guidelines. We utilized 2^−∆∆CT^ to calculate the gene expression level difference between the wild type and mutant, and 40S and α-ACTIN genes were used as controls [38]. Duncan’s test evaluated significant differences between samples for all experimental data.

### Yeast two-hybrid and BiFC assay

Yeast two-hybrid (Y2H) system used Y2H system kits (Coolaber, China). The yeast strain Y2H-Gold was used in this system, in which the AD vector was pGADT7, and the BD vector was pGBKT7. The positive controls were pGADT7-T and pGBKT7-53. The assays were performed according to the protocol provided with the kit. For BiFC assay, the fragments of MoCdc28, MoMps1, MoAtg17 and MoAtg18 were cloned into pKD5-YFPC vector to generate MoCdc28-YFPC, MoMps1-YFPC, MoAtg17-YFPC and MoATG18-YFPC fusion constructs, respectively. MoSwe1-YFPN constructs were generated by cloning the MoSwe1 fragments into the pKD2-YFPN vector. A pair of MoSwe1-YFPN and MoCdc28-YFPC (MoMps1-YFPC, MoAtg17-YFPC, MoATG18-YFPC) constructs were cotransformed into 70-15 by ATMT. Transformants resistant to both sulfonylurea and hygromycin were isolated. YFP signals were detected using a LSM880 confocal microscope.

### In vitro pull-down assays

MoSwe1-N2 was amplified from the cDNA of MoSwe1 and ligated to the vector PGEX4T-1 to obtain the MoSwe1-N2-GST plasmid. MoAtg17 and MoAtg18 were amplified from the cDNA of *M. oryzae* and ligated to the vector pET-32a to obtain the MoAtg17-His and MoAtg18-His plasmids. The MoSwe1-N2-GST, MoAtg17-His, and MoAtg18-His plasmids were transformed into the expression *E. coli* strain (DE3 + Plyse), IPTG (4mM) was added, and the expression of the three proteins was detected after overnight culture at 18°C. GST magnetic beads were used to pull MoSwe1-N2-GST protein, which was enriched on the magnetic beads, and MoAtg17-His or MoAtg18-His were added and eluted. MoAtg17-His or MoAtg18-His was detected by an anti-His antibody in an elution buffer (HuaBio, China).

### Western blot assays

The fungal hyphae were shaken using liquid CM for two days and then transferred to SD-N medium (0.17% yeast nitrogen base without amino acids and ammonium sulfate, 2% glucose) for an additional 4 h of nitrogen starvation induction to triggering autophagy. Western blot detection of GFP-MoAtg8 fusion protein with GFP antibody (GFP 1:10,000-20000; Abcam). To detect endogenous MoAtg8/MoAtg8-PE, 70-15 and the Δ*Moswe1* mutant were cultivated in liquid CM for 48 h and then rapidly transferred to SD-N medium for 3 or 6 h. The protein was extracted by the TCA method, and an anti-Atg8 antibody (Atg8 1:500-1,000; MBL) was used to detect MoAtg8 and MoAtg8-PE levels.

Total proteins were isolated by TCA-acetone methods to assay the phosphorylated Mps1 levels. We used an anti-P-p44/42 antibody (Cell Signalling Technology) and an anti-p44/42 MAPK antibody (Cell Signalling Technology) as controls. Total proteins were extracted from hyphae grown in liquid CM to detect phosphorylated Cdc2. We used an anti-cdc2 antibody (Santa Cruz Biotechnology) and Phospho-cdc2 (Tyr15) antibody (Cell Signalling Technology) to detect nonphosphorylated and phosphorylated Cdc28 levels, respectively.

### Supplementary Information

**Additional file 1: Fig. S1.** Alignment and comparison of Swe1 and its homologues in many eukaryotes and Swe1 protein structure. **Fig. S2.** Establishment of gene deletion and identification of knockout mutants. **Fig. S3.** The Δ*MoSwe1* mutant is not sensitive to the DNA replication inhibitor HU and cell cycle for appressorium formation at 24 hpi in *M. oryzae*. **Fig. S4.** Δ*Moswe1-N2-C* strain affects virulence, and MoSwe1 influences the localization of the septin ring in *M. oryzae*. **Fig. S5.** Glycogen and lipid body utilization and degradation in the Δ*Moswe1* mutant. **Fig. S6.** Metabolomics and transcriptomics analysis indicates that MoSwe1 participates in glycogen and fatty acid metabolism and the autophagy pathway. **Table S1.** Primers in this study.

## Data Availability

Dataset used or analyzed during the current study are available from the corresponding author on reasonable request.

## References

[1] Asano S, Park JE, Sakchaisri K, Yu LR, Song S, Supavilai P, Veenstra TD, Lee KS (2005). Concerted mechanism of Swe1/Wee1 regulation by multiple kinases in budding yeast. EMBO J.

[2] Badaruddin M, Holcombe LJ, Wilson RA, Wang ZY, Kershaw MJ, Talbot NJ. Glycogen metabolic genes are involved in trehalose-6-phosphate synthase-mediated regulation of pathogenicity by the rice blast fungus Magnaporthe oryzae. PLoS Pathog. 2013;9:e1003604. 10.1371/journal.ppat.1003604 PMC3789717 24098112

[3] Barral Y, Parra M, Bidlingmaier S, Snyder M (1999). Nim1-related kinases coordinate cell cycle progression with the organization of the peripheral cytoskeleton in yeast. Genes Dev.

[4] Besson A, Dowdy SF, Roberts JM (2008). CDK inhibitors: cell cycle regulators and beyond. Dev Cell.

[5] Bhadauria V, Banniza S, Vandenberg A, Selvaraj G, Wei Y (2012). Peroxisomal alanine: glyoxylate aminotransferase AGT1 is indispensable for appressorium function of the rice blast pathogen, Magnaporthe oryzae. PLoS ONE.

[6] Bhambra GK, Wang ZY, Soanes DM, Wakley GE, Talbot NJ (2006). Peroxisomal carnitine acetyl transferase is required for elaboration of penetration hyphae during plant Infection by Magnaporthe Grisea. Mol Microbiol.

[7] Booher RN, Deshaies RJ, Kirschner MW (1993). Properties of Saccharomyces cerevisiae wee1 and its differential regulation of p34CDC28 in response to G1 and G2 cyclins. EMBO J.

[8] Cao H, Huang P, Zhang L, Shi Y, Sun D, Yan Y, Liu X, Dong B, Chen G, Snyder JH (2016). Characterization of 47 Cys2 -His2 zinc finger proteins required for the development and pathogenicity of the rice blast fungus Magnaporthe oryzae. New Phytol.

[9] Carroll CW, Altman R, Schieltz D, Yates JR, Kellogg D (1998). The septins are required for the mitosis-specific activation of the Gin4 kinase. J Cell Biol.

[10] Cools T, De Veylder L (2009). DNA stress checkpoint control and plant development. Curr Opin Plant Biol.

[11] Dagdas YF, Yoshino K, Dagdas G, Ryder LS, Bielska E, Steinberg G, Talbot NJ (2012). Septin-mediated plant cell invasion by the rice blast fungus, Magnaporthe oryzae. Science.

[12] Dalby KN, Morrice N, Caudwell FB, Avruch J, Cohen P (1998). Identification of regulatory phosphorylation sites in mitogen-activated protein kinase (MAPK)-activated protein kinase-1a/p90rsk that are inducible by MAPK. J Biol Chem.

[13] Feng W, Yin Z, Wu H, Liu P, Liu X, Liu M, Yu R, Gao C, Zhang H, Zheng X (2021). Balancing of the mitotic exit network and cell wall integrity signaling governs the development and pathogenicity in Magnaporthe oryzae. PLoS Pathog.

[14] Fernandez J, Marroquin-Guzman M, Wilson RA (2014). Evidence for a transketolase-mediated metabolic checkpoint governing biotrophic growth in rice cells by the blast fungus Magnaporthe oryzae. PLoS Pathog.

[15] Flor-Parra I, Vranes M, Kamper J, Perez-Martin J (2006). Biz1, a zinc finger protein required for plant invasion by Ustilago maydis, regulates the levels of a mitotic cyclin. Plant Cell.

[16] Fukada F, Kubo Y (2015). Colletotrichum orbiculare regulates cell cycle G1/S progression via a two-component GAP and a GTPase to establish plant Infection. Plant Cell.

[17] Fukada F, Kodama S, Nishiuchi T, Kajikawa N, Kubo Y (2019). Plant pathogenic fungi Colletotrichum and Magnaporthe share a common G(1) phase monitoring strategy for proper appressorium development. New Phytol.

[18] Haase SB, Wittenberg C (2014). Topology and control of the cell-cycle-regulated transcriptional circuitry. Genetics.

[19] Harris SD, Morrell JL, Hamer JE (1994). Identification and characterization of aspergillus nidulans mutants defective in cytokinesis. Genetics.

[20] Harvey SL, Kellogg DR (2003). Conservation of mechanisms controlling entry into mitosis: budding yeast wee1 delays entry into mitosis and is required for cell size control. Curr Biol.

[21] Harvey SL, Charlet A, Haas W, Gygi SP, Kellogg DR. Cdk1-dependent regulation of the mitotic inhibitor Wee1. Cell. 2005;122:407–420. 10.1016/j.cell.2005.05.029 16096060

[22] He M, Kershaw MJ, Soanes DM, Xia Y, Talbot NJ (2012). Infection-associated nuclear degeneration in the rice blast fungus Magnaporthe Oryzae requires non-selective macro-autophagy. PLoS ONE.

[23] Hood-DeGrenier JK, Boulton CN, Lyo V (2007). Cytoplasmic Clb2 is required for timely inactivation of the mitotic inhibitor Swe1 and normal bud morphogenesis in Saccharomyces cerevisiae. Curr Genet.

[24] Howard RJ, Valent B (1996). Breaking and entering: host penetration by the fungal rice blast pathogen Magnaporthe Grisea. Annu Rev Microbiol.

[25] Jeon J, Goh J, Yoo S, Chi MH, Choi J, Rho HS, Park J, Han SS, Kim BR, Park SY (2008). A putative MAP kinase kinase kinase, MCK1, is required for cell wall integrity and pathogenicity of the rice blast fungus, Magnaporthe oryzae. Mol Plant Microbe Interact.

[26] Jiang C, Zhang X, Liu H, Xu JR (2018). Mitogen-activated protein kinase signaling in plant pathogenic fungi. PLoS Pathog.

[27] Kellogg DR (2003). Wee1-dependent mechanisms required for coordination of cell growth and cell division. J Cell Sci.

[28] Kershaw MJ, Talbot NJ (2009). Genome-wide functional analysis reveals that infection-associated fungal autophagy is necessary for rice blast Disease. Proc Natl Acad Sci U S A.

[29] Kipreos ET (2005). C. Elegans cell cycles: invariance and stem cell divisions. Nat Rev Mol Cell Biol.

[30] Kono K, Nogami S, Abe M, Nishizawa M, Morishita S, Pellman D, Ohya Y (2008). G1/S cyclin-dependent kinase regulates small GTPase Rho1p through phosphorylation of RhoGEF Tus1p in Saccharomyces cerevisiae. Mol Biol Cell.

[31] Lee KS, Irie K, Gotoh Y, Watanabe Y, Araki H, Nishida E, Matsumoto K, Levin DE (1993). A yeast mitogen-activated protein kinase homolog (Mpk1p) mediates signalling by protein kinase C. Mol Cell Biol.

[32] Levin DE (2005). Cell wall integrity signaling in Saccharomyces cerevisiae. Microbiol Mol Biol Rev.

[33] Levine B, Kroemer G (2019). Biological functions of Autophagy genes: a Disease Perspective. Cell.

[34] Liu H, Wang Y (2006). The function and regulation of budding yeast Swe1 in response to interrupted DNA synthesis. Mol Biol Cell.

[35] Liu XH, Lu JP, Lin FC. Autophagy during conidiation, conidial germination and turgor generation in Magnaporthe grisea. Autophagy. 2007;3:472–473. 10.4161/auto.4339 17495517

[36] Liu XH, Lu JP, Zhang L, Dong B, Min H, Lin FC (2007). Involvement of a Magnaporthe grisea serine/threonine kinase gene, MgATG1, in appressorium turgor and pathogenesis. Eukaryot Cell.

[37] Liu XH, Xu F, Snyder JH, Shi HB, Lu JP, Lin FC (2016). Autophagy in plant pathogenic fungi. Semin Cell Dev Biol.

[38] Livak KJ, Schmittgen TD (2001). Analysis of relative gene expression data using real-time quantitative PCR and the 2(-Delta Delta C(T)) Method.

[39] Longtine MS, Theesfeld CL, McMillan JN, Weaver E, Pringle JR, Lew DJ (2000). Septin-dependent assembly of a cell cycle-regulatory module in Saccharomyces cerevisiae. Mol Cell Biol.

[40] Lu J, Cao H, Zhang L, Huang P, Lin F (2014). Systematic analysis of Zn2Cys6 transcription factors required for development and pathogenicity by high-throughput gene knockout in the rice blast fungus. PLoS Pathog.

[41] Ma XJ, Lu Q, Grunstein M (1996). A search for proteins that interact genetically with histone H3 and H4 amino termini uncovers novel regulators of the Swe1 kinase in Saccharomyces cerevisiae. Genes Dev.

[42] Malavazi I, Goldman GH, Brown NA (2014). The importance of connections between the cell wall integrity pathway and the unfolded protein response in filamentous fungi. Brief Funct Genomics.

[43] Malumbres M, Barbacid M (2009). Cell cycle, CDKs and cancer: a changing paradigm. Nat Rev Cancer.

[44] Marroquin-Guzman M, Sun G, Wilson RA (2017). Glucose-ABL1-TOR signaling modulates cell cycle tuning to Control Terminal Appressorial Cell differentiation. PLoS Genet.

[45] Martin H, Arroyo J, Sanchez M, Molina M, Nombela C (1993). Activity of the yeast MAP kinase homologue Slt2 is critically required for cell integrity at 37 degrees C. Mol Gen Genet.

[46] Martin-Urdiroz M, Oses-Ruiz M, Ryder LS, Talbot NJ (2016). Investigating the biology of plant Infection by the rice blast fungus Magnaporthe oryzae. Fungal Genet Biol.

[47] McMillan JN, Longtine MS, Sia RA, Theesfeld CL, Bardes ES, Pringle JR, Lew DJ (1999). The morphogenesis checkpoint in Saccharomyces cerevisiae: cell cycle control of Swe1p degradation by Hsl1p and Hsl7p. Mol Cell Biol.

[48] McMillan JN, Theesfeld CL, Harrison JC, Bardes ES, Lew DJ (2002). Determinants of Swe1p degradation in Saccharomyces cerevisiae. Mol Biol Cell.

[49] Mercer TJ, Ohashi Y, Boeing S, Jefferies HBJ, De Tito S, Flynn H, Tremel S, Zhang W, Wirth M, Frith D et al. Phosphoproteomic identification of ULK substrates reveals VPS15-dependent ULK/VPS34 interplay in the regulation of autophagy. EMBO J. 2021;40:e105985. 10.15252/embj.2020105985 PMC8280838 34121209

[50] Moffat J, Andrews B (2003). Ac’septin’ a signal: kinase regulation by septins. Dev Cell.

[51] Nurse P (1975). Genetic control of cell size at cell division in yeast. Nature.

[52] Oses-Ruiz M, Talbot NJ (2017). Cell cycle-dependent regulation of plant Infection by the rice blast fungus Magnaporthe oryzae. Commun Integr Biol.

[53] Oses-Ruiz M, Sakulkoo W, Littlejohn GR, Martin-Urdiroz M, Talbot NJ. Two Independent S-phase checkpoints regulate appressorium-mediated plant Infection by the rice blast fungus Magnaporthe oryzae. Proc Natl Acad Sci USA. 2017;114:E237–44. 10.1073/pnas.1611307114 PMC5240714 28028232

[54] Parker D, Beckmann M, Enot DP, Overy DP, Rios ZC, Gilbert M, Talbot N, Draper J (2008). Rice blast Infection of Brachypodium distachyon as a model system to study dynamic host/pathogen interactions. Nat Protoc.

[55] Patkar RN, Ramos-Pamplona M, Gupta AP, Fan Y, Naqvi NI (2012). Mitochondrial beta-oxidation regulates organellar integrity and is necessary for conidial germination and invasive growth in Magnaporthe oryzae. Mol Microbiol.

[56] Perfect SE, Hughes HB, O’Connell RJ, Green JR (1999). Colletotrichum: a model genus for studies on pathology and fungal-plant interactions. Fungal Genet Biol.

[57] Ramos-Pamplona M, Naqvi NI (2006). Host invasion during rice-blast Disease requires carnitine-dependent transport of peroxisomal acetyl-CoA. Mol Microbiol.

[58] Roux PP, Blenis J (2004). ERK and p38 MAPK-activated protein kinases: a family of protein kinases with diverse biological functions. Microbiol Mol Biol Rev.

[59] Russell P, Moreno S, Reed SI (1989). Conservation of mitotic controls in fission and budding yeasts. Cell.

[60] Sakchaisri K, Asano S, Yu LR, Shulewitz MJ, Park CJ, Park JE, Cho YW, Veenstra TD, Thorner J, Lee KS (2004). Coupling morphogenesis to mitotic entry. Proc Natl Acad Sci U S A.

[61] Saunders DG, Aves SJ, Talbot NJ (2010). Cell cycle-mediated regulation of plant Infection by the rice blast fungus. Plant Cell.

[62] Saunders DG, Dagdas YF, Talbot NJ (2010). Spatial uncoupling of mitosis and cytokinesis during appressorium-mediated plant Infection by the rice blast fungus Magnaporthe oryzae. Plant Cell.

[63] Sia RA, Bardes ES, Lew DJ (1998). Control of Swe1p degradation by the morphogenesis checkpoint. EMBO J.

[64] Talbot NJ (2003). On the trail of a cereal killer: exploring the biology of Magnaporthe Grisea. Annu Rev Microbiol.

[65] Tanida I (2011). Autophagosome formation and molecular mechanism of autophagy. Antioxid Redox Signal.

[66] Thery M, Bornens M (2006). Cell shape and cell division. Curr Opin Cell Biol.

[67] Thines E, Weber RW, Talbot NJ (2000). MAP kinase and protein kinase A-dependent mobilization of triacylglycerol and glycogen during appressorium turgor generation by Magnaporthe Grisea. Plant Cell.

[68] Veneault-Fourrey C, Barooah M, Egan M, Wakley G, Talbot NJ (2006). Autophagic fungal cell death is necessary for Infection by the rice blast fungus. Science.

[69] Voigt O, Poggeler S (2013). Self-eating to grow and kill: autophagy in filamentous ascomycetes. Appl Microbiol Biotechnol.

[70] Walczak M, Martens S (2013). Dissecting the role of the Atg12-Atg5-Atg16 complex during autophagosome formation. Autophagy.

[71] Walter SA, Guadagno SN, Ferrell JE (2000). Activation of Wee1 by p42 MAPK in vitro and in cycling xenopus egg extracts. Mol Biol Cell.

[72] Wang ZY, Thornton CR, Kershaw MJ, Debao L, Talbot NJ (2003). The glyoxylate cycle is required for temporal regulation of virulence by the plant pathogenic fungus Magnaporthe Grisea. Mol Microbiol.

[73] Wang ZY, Soanes DM, Kershaw MJ, Talbot NJ (2007). Functional analysis of lipid metabolism in Magnaporthe Grisea reveals a requirement for peroxisomal fatty acid beta-oxidation during appressorium-mediated plant Infection. Mol Plant Microbe Interact.

[74] Widmann C, Gibson S, Jarpe MB, Johnson GL (1999). Mitogen-activated protein kinase: conservation of a three-kinase module from yeast to human. Physiol Rev.

[75] Wilson RA, Talbot NJ (2009). Under pressure: investigating the biology of plant Infection by Magnaporthe oryzae. Nat Rev Microbiol.

[76] Xie Z, Klionsky DJ (2007). Autophagosome formation: core machinery and adaptations. Nat Cell Biol.

[77] Xu JR, Staiger CJ, Hamer JE (1998). Inactivation of the mitogen-activated protein kinase Mps1 from the rice blast fungus prevents penetration of host cells but allows activation of plant defense responses. Proc Natl Acad Sci U S A.

[78] Yin Z, Tang W, Wang J, Liu X, Yang L, Gao C, Zhang J, Zhang H, Zheng X, Wang P (2016). Phosphodiesterase MoPdeH targets MoMck1 of the conserved mitogen-activated protein (MAP) kinase signalling pathway to regulate cell wall integrity in rice blast fungus Magnaporthe oryzae. Mol Plant Pathol.

[79] Yin Z, Feng W, Chen C, Xu J, Li Y, Yang L, Wang J, Liu X, Wang W, Gao C (2020). Shedding light on autophagy coordinating with cell wall integrity signaling to govern pathogenicity of Magnaporthe oryzae. Autophagy.

[80] Zhu XM, Li L, Wu M, Liang S, Shi HB, Liu XH, Lin FC (2019). Current opinions on autophagy in pathogenicity of fungi. Virulence.

