# Supplementary material for: The cell cycle, autophagy, and cell wall integrity pathway jointly governed by MoSwe1 in Magnaporthe oryzae
Source: Cell Commun Signal. 2024 Jan 9;22:19. doi: 10.1186/s12964-023-01389-6 (PMC10775494; doi:10.1186/s12964-023-01389-6)
Supplement: Supplementary file 2 — Additional file 1: Fig. S1. Alignment and comparison of Swe1 and its homologues in many eukaryotes and Swe1 protein structure. Fig. S2. Establishment of gene deletion and identification of knockout mutants. Fig. S3. The ΔMoSwe1 mutant is not sensitive to the DNA replication inhibitor HU and cell cycle for appressorium formation at 24 hpi in M. oryzae. Fig. S4. ΔMoswe1-N2-C strain affects virulence, and MoSwe1 influences the localization of the septin ring in M. oryzae. Fig. S5. Glycogen and lipid body utilization and degradation in the ΔMoswe1 mutant. Fig. S6. Metabolomics and transcriptomics analysis indicates that MoSwe1 participates in glycogen and fatty acid metabolism and the autophagy pathway. Table S1. Primers in this study. [file 12964_2023_1389_MOESM1_ESM.docx]

**Supplementary information**

**Cell cycle, autophagy, and cell wall integrity pathway jointly govern the pathogenicity of *Magnaporthe oryzae***

Lin Li ^a^, Xue-Ming Zhu ^a^, Jian-Dong Bao ^a^, Jiao-Yu Wang ^a^, Xiao-Hong Liu ^b^, Fu-Cheng Lin ^a, b*^

^a^ State Key Laboratory for Managing Biotic and Chemical Treats to the Quality and Safety of Agro-products, Institute of Plant Protection and Microbiology, Zhejiang Academy of Agricultural Sciences, Hangzhou, 310021, China.

^b^ Institute of Biotechnology, Zhejiang University, Hangzhou, 310058, China.

*Corresponding author: Tel: +86 571 88404007; E-mail: [fuchenglin@zju.edu.cn](mailto:fuchenglin@zju.edu.cn)


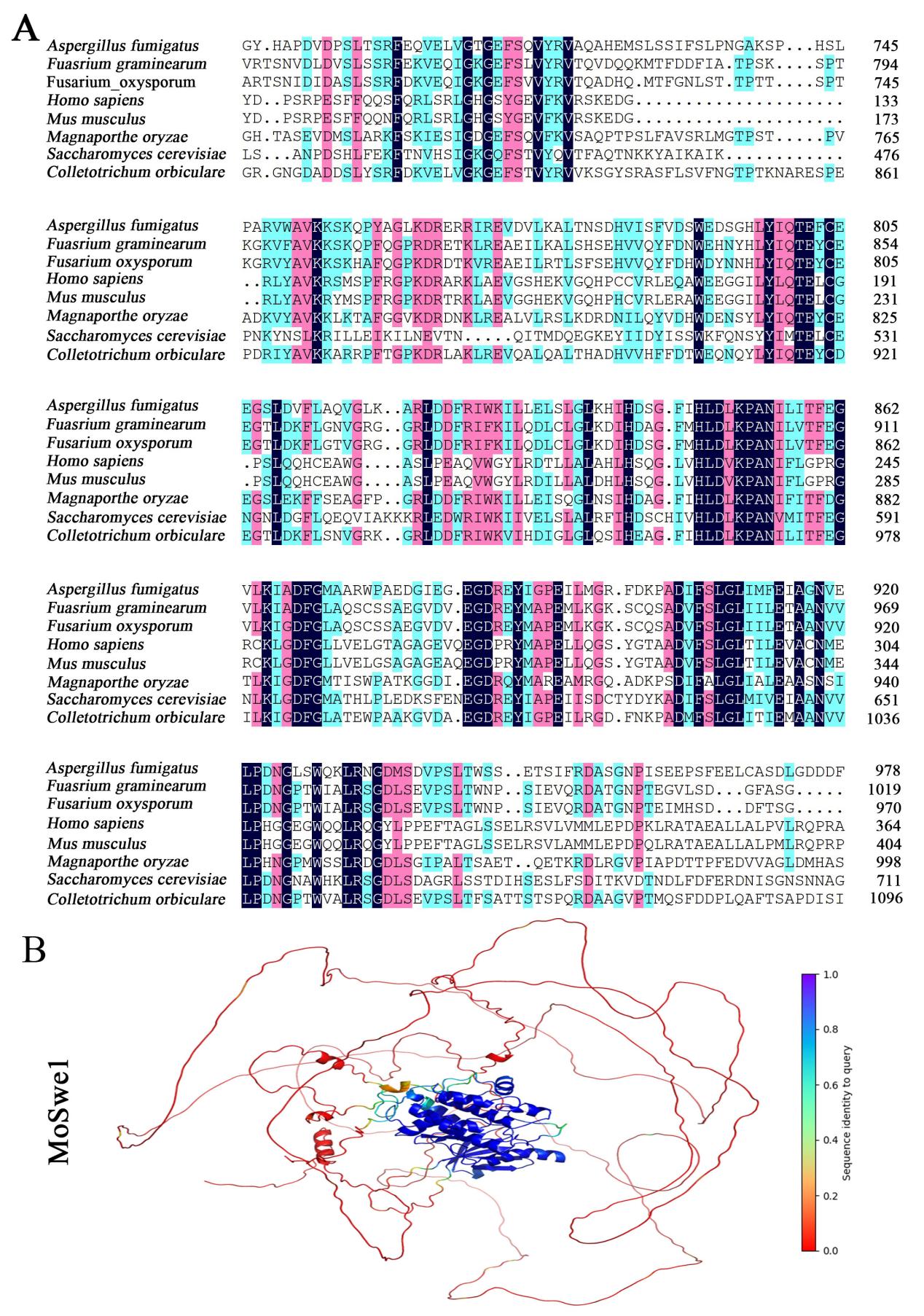


**Fig. S1. Alignment and comparison of Swe1 and its homologues in many eukaryotes and Swe1 protein structure**

A. Alignment of the amino acids of Swe1 in *M. oryzae* (MoSwe1) with those of the homologous proteins in *C. orbiculare* (TDZ26209.1), *Fusarium graminearum* (PCD18808.1), *Aspergillus fumigatus* (KEY81105.1), *Saccharomyces cerevisiae* (NP_012348.1), *Fusarium oxysporum* (RKK76988.1), *Mus musculus* (XP_006524373.1) and *Homo sapiens* (AAI21162.1) by CLUSTALW.

B. The structure confidence of MoSwe1 (score = 50.1) by the coloured polymers based on pLDDT.

**
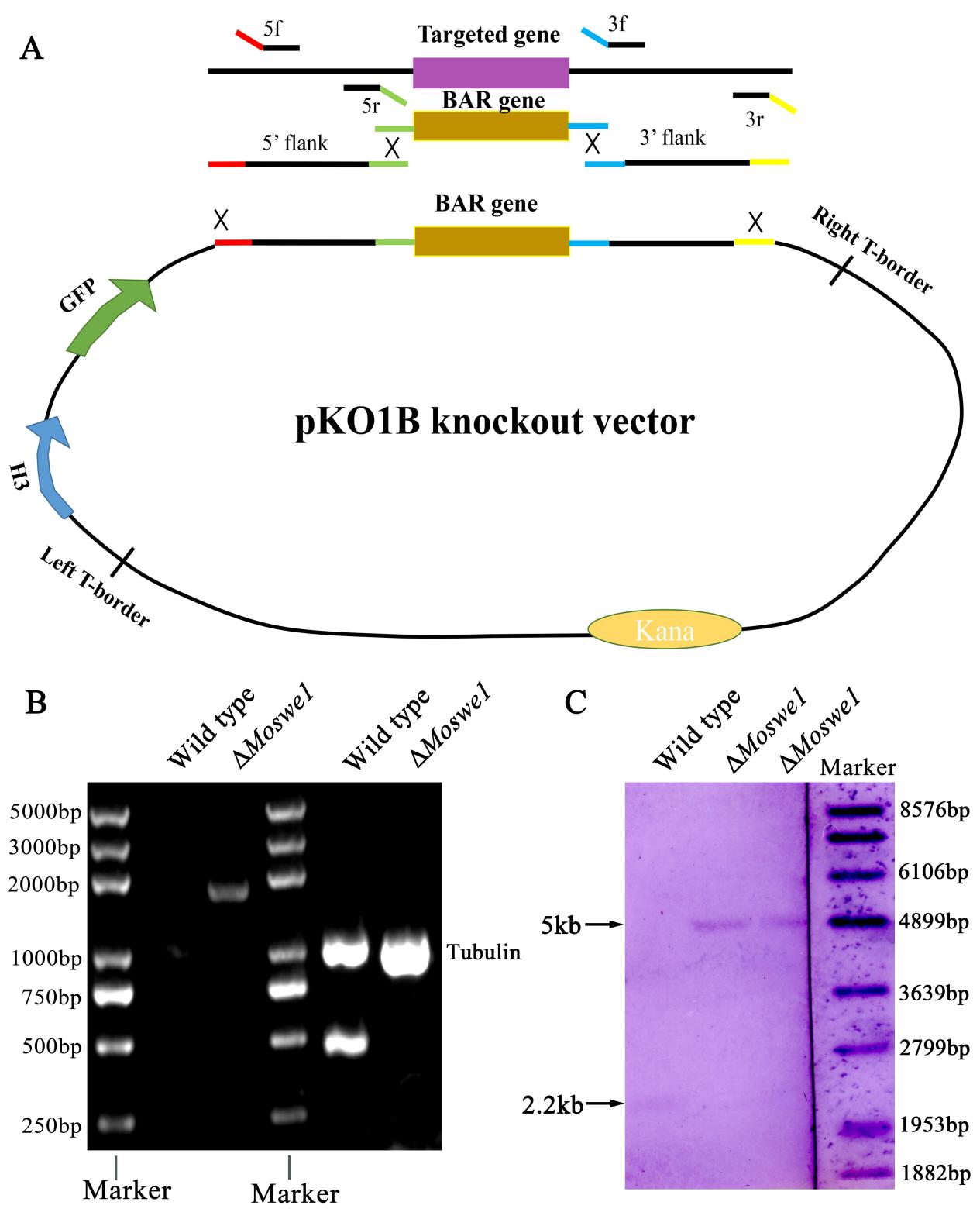
**

**Fig. S2. Establishment of gene deletion and identification of knockout mutants**

A. Schematic diagram of constructing PKO1B knockout vector.

B. PCR screened the targeted gene in the transformant, and tubulin genes of *M. oryzae* were used as control. A 2.0 kb band was amplified from the Δ*Moswe1* mutant, while the wild type 70-15 did not exhibit the band (left). A characteristic band (500 bp) can be detected in the wild type 70-15, indicating that the target gene was present, while the band was not found in the Δ*Moswe1* mutant. Tubulin bands (1.0 kb) act as positive controls.

C. Wild type 70-15 and Δ*Moswe1* mutant were verified by Southern blot. Genomic DNA was digested with *Pst*I. The DNA was hybridized with a 1.0 kb fragment amplified from genomic DNA of *M. oryzae* wild-type strain 70-15.


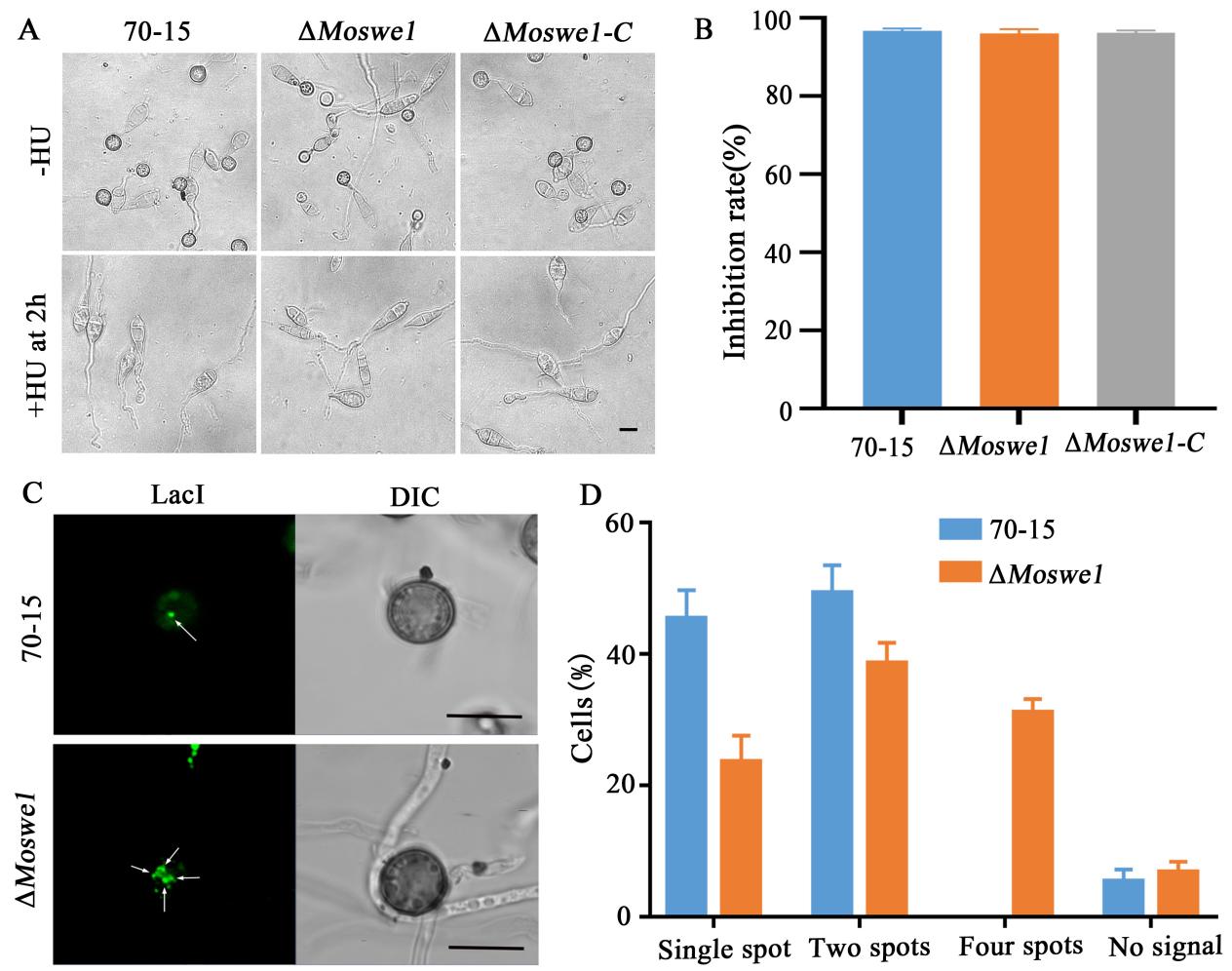


**Fig. S3. The Δ*MoSwe1* mutant is not sensitive to the DNA replication inhibitor HU and cell cycle for appressorium formation at 24 hpi in *M. oryzae***

A. Sensitivity test of the DNA replication inhibitor HU. Scale bar, 10 μm.

B. The inhibition rate was calculated using the number of appressoria formed without HU as a control.

C. Representative images of LacO/LacI-GFP-transformed strains of wild-type 70-15 and the Δ*Moswe1* mutant during appressorium formation after 24 h of incubation. Scale bar, 10 μm.

D. Average percentage of the appressorium cell cycle progression in the wild type 70-15 and the Δ*Moswe1* mutant.

**
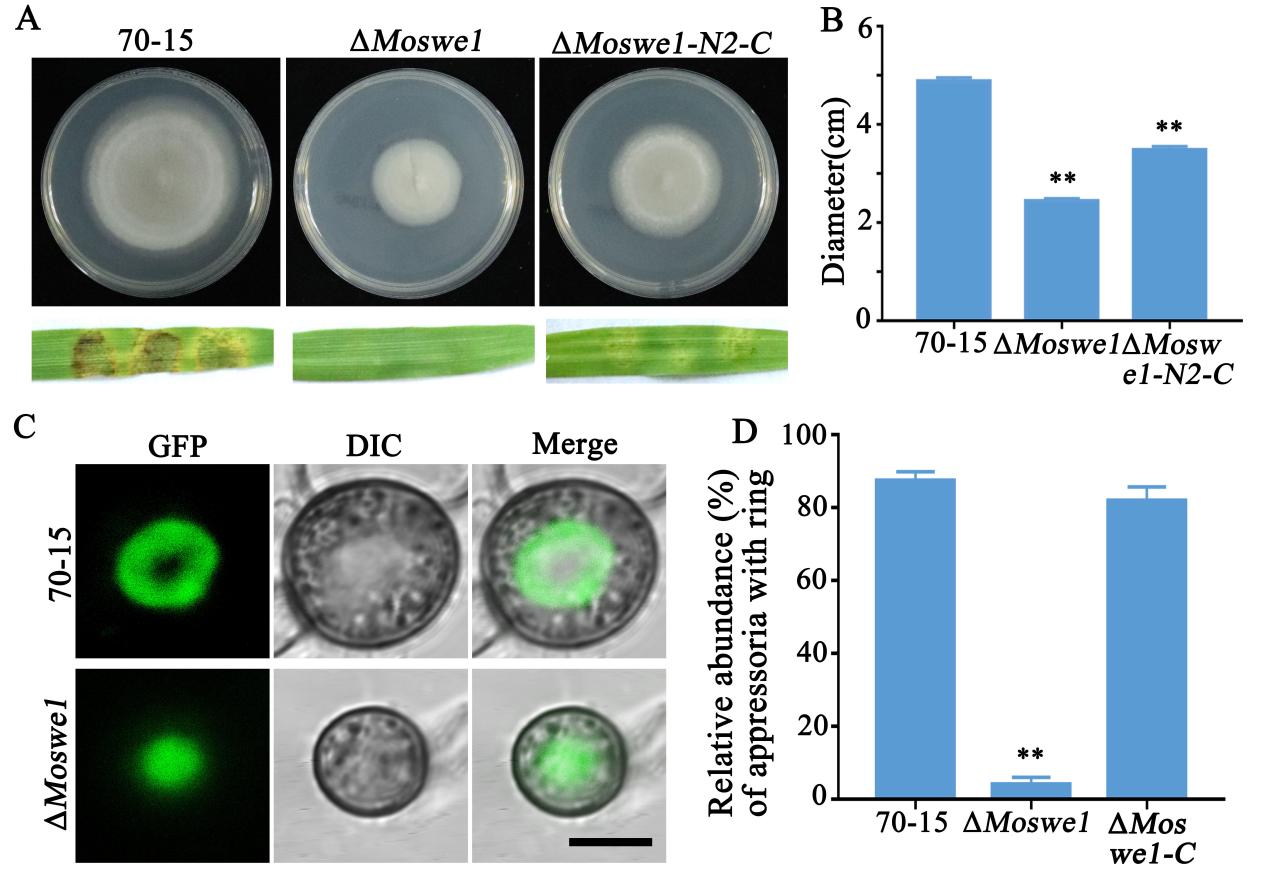
**

**Fig. S4.** **Δ*Moswe1-N2*-*C* strain affects virulence, and MoSwe1 influences the localization of the septin ring in *M. oryzae***

A. 70-15, Δ*Moswe1* and the Δ*Moswe1-N2-C* strains were grown on CM medium for 8 days, and pathogenicity was detected.

B. Diameters of colonies (Duncan’s test, *P* < 0.01).

C. Typical pictures about the morphology of septin ring at the appressorium pore in 70-15 and Δ*Moswe1* mutant. 70-15 containing MoSep5-GFP was induced on hydrophobic film for 24 h to generate mature appressoria: scale bar, 5 μm.

D. Average proportion of conidia forming normal a septin ring. At least 60 appressoria were counted per experiment (Duncan’s test, *P* < 0.01).

**
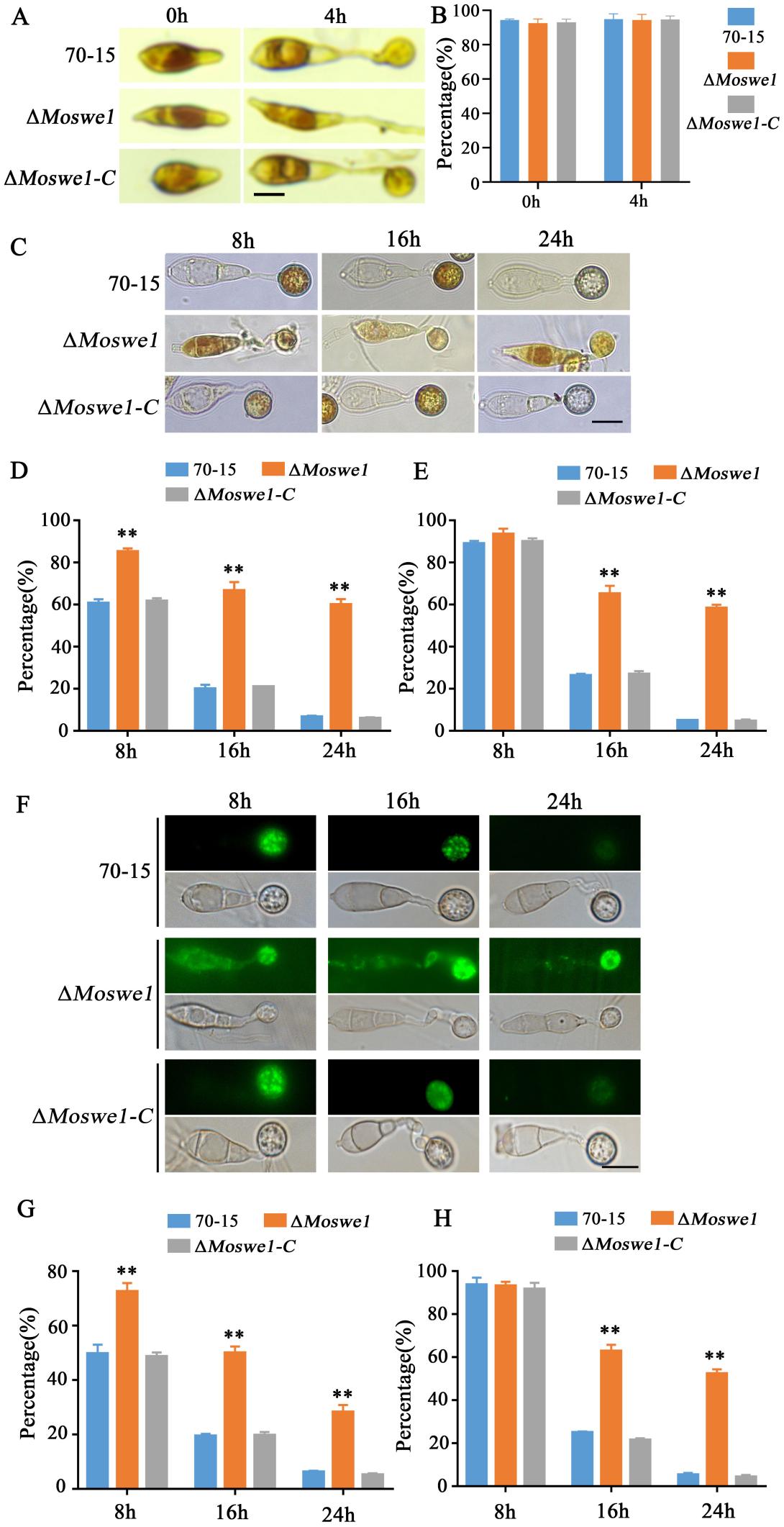
**

**Fig. S5. Glycogen and lipid body utilization and degradation in the Δ*Moswe1* mutant**

A and C. The glycogen in the conidium and appressorium was stained burgundy with KI/I_2_ solution at regular intervals, and the distribution of glycogen was observed with a microscope: scale bar, 10 μm.

B and D. The ratio of glycogen in conidia.

E. The proportion of glycogen in appressoria during appressorium development.

F. Lipid bodies in the conidium and appressorium were stained green with BODIPY solution at regular intervals, and the distribution of lipid bodies was observed with a microscope: scale bar, 10 μm.

G. The ratio of lipid bodies in conidia.

H. The proportion of lipid bodies in appressoria during appressorium development.

**
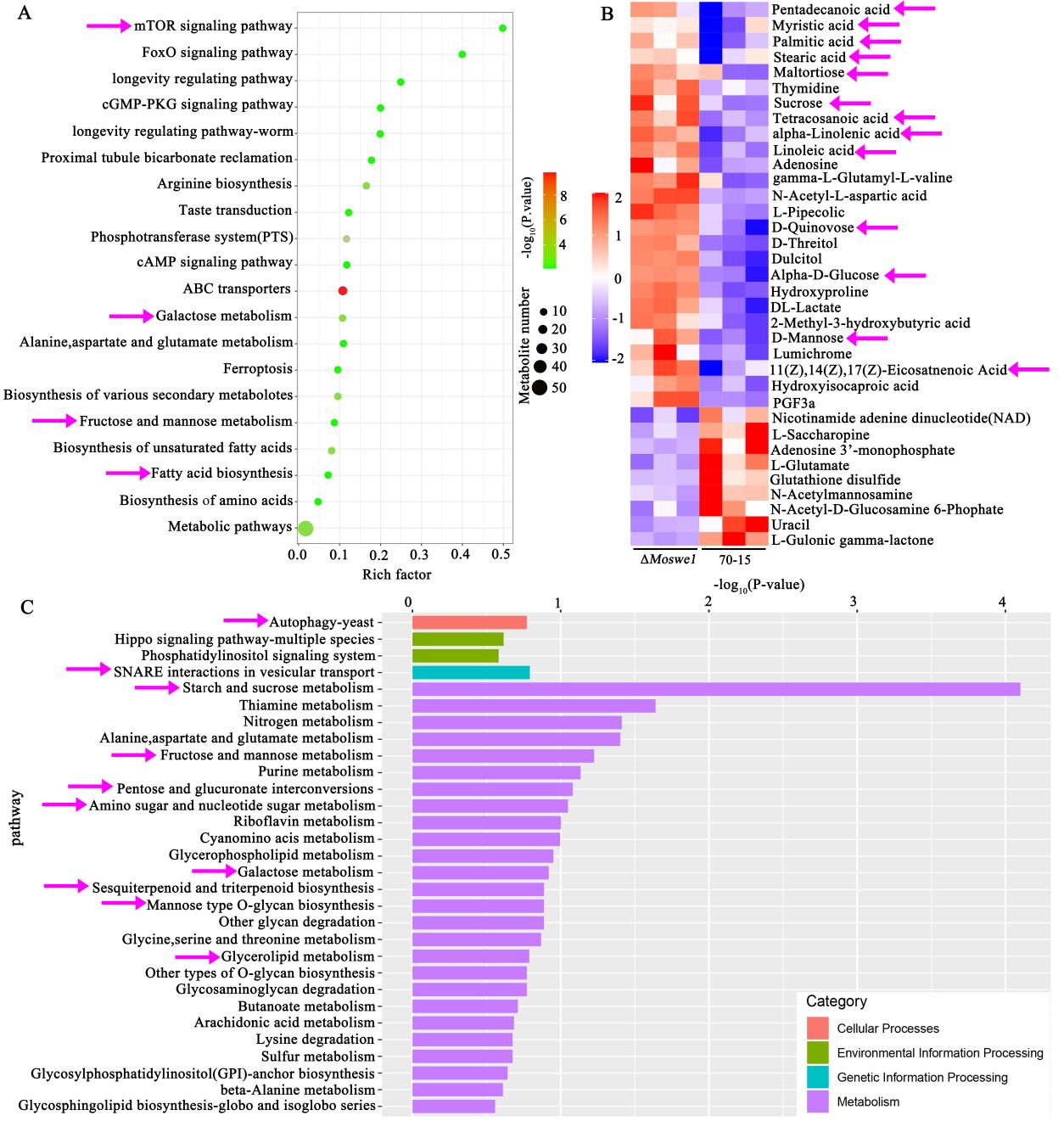
**

**Fig. S6.** **Metabolomics and transcriptomics analysis indicates that MoSwe1 participates in glycogen and fatty acid metabolism and the autophagy pathway**

A. Comparing the differences in KEGG analysis results of 70-15 and Δ*Moswe1* samples.

The data manifested significant enrichment of many pathways through metabolomics.

B. Heat map analysis of 70-15 and Δ*Moswe1* metabolomics differences.

C. Comparing the differences in KEGG analysis results of 70-15 and Δ*Moswe1*. The results proved significant enrichment of numerous pathways through transcriptomics.

**Table S1 Primers in this study**

| Name Sequence (5’ - 3’) | |
| --- | --- |
| Primers used for gene knockout | |
| BAR-F | AGAAGATGATATTGAAGGAGC |
| BAR-R | CTAAATCTCGGTGACGGGCAG |
| MoSWE1up-F | CCGGGGATCCTCTAGAAACAAACGCGACTTCCTT |
| MoSWE1up-R | TGTTGACCTCCACTATGCGGCTGAATGCAGTTG |
| MoSWE1dn-F | GGAATAGAGTAGATGCTGCCAGAAGCTTGTTTGGAG |
| MoSWE1dn-R | GGCCAGTGCCAAGCTTTTGCATTCATCACAAAGCG |
| MoSWE1inner-F | GCCATCAAAATTCCTGCTGCG |
| MoSWE1inner-R | TTTGGCTCATCACCGTTGAGCG |
| MoSWE1upyz-F | GCACCTTTTGAGAATCCC |
| MoSWE1upyz-R | AGGGCGAACTTAAGAAGGTATG |
| Primers used for complementation | |
| pKD5-MoSWE1-F | CACAATCACTAGTGAATTCAAGCCTCCGAATGTCTCT |
| pKD5-MoSWE1-R | CATCCCGGGGATGGATCCAACGTCGGTCATCTCGACGT |
| Primer used for Real-Time PCR | |
| qRT-tublin-F | ACAACTTCGTCTTCGGTCAG |
| qRT-tublin-R | GTGATCTGGAAACCCTGGAG |
| qRT-BAR-F | CACCATCGTCAACCACTACATC |
| qRT-BAR-R | GCGACGAGCCAGGGATA |
| qRT-SWE1-F | CGGATCTGGAAGATTCTGCTC |
| qRT-SWE1-R | TCAGAGTGCCATCAAAGGTG |
| Primer used for Pull-down | |
| GST-SWE1-N2-F | CCGCGTGGATCCCCGGAATTCCGCGATATCGCGCCTCCACCA |
| GST-SWE1-N2-R | CTCGAGTCGACCCGGGAATTCCTTCCTTGCCAAACTCATATC |
| His-ATG17-F | CAAGGTCGACAAGCTTATGCCGTCTTCAAGTTCCGCC |
| His-ATG17-R | GTGCGGCCGCAAGCTTACGTCCATGTACCCTCTCCCT |
| His-ATG18-F | CAAGGTCGACAAGCTTATGGCGACTGCAACGCTAAAC |
| His-ATG18-R | GTGCGGCCGCAAGCTTAGACTCATATGTCGAAGAAGA |
| Primer used for two-hybrid | |
| AD-SWE1-N2-F | GGAGGCCAGTGAATTCCGCGATATCGCGCCTCCACCA |
| AD-SWE1-N2-R | CGAGCTCGATGGATCCCTTCCTTGCCAAACTCATATC |
| BD-ATG17-F | GGAGGCCAGTGAATTCATGCCGTCTTCAAGTTCCGCC |
| BD-ATG17-R | CGAGCTCGATGGATCCACGTCCATGTACCCTCTCCCT |
| BD-ATG18-F | GGAGGCCAGTGAATTCATGGCGACTGCAACGCTAAAC |
| BD-ATG18-R | CGAGCTCGATGGATCCAGACTCATATGTCGAAGAAGA |
| BD-CDC28-F | CATGGAGGCCGAATTCATGGAAAACTACCAGAAGCTC |
| BD-CDC28-R | GCAGGTCGACGGATCCTCGCCTCGGGGGCGCTGGGGTGT |
| Primer used for BiFC | |
| ATG17-YFPC-F | ATCACAATGGCCGGATCCATGCCGTCTTCAAGTTCCGCC |
| ATG17-YFPC-R | GCAGGCCGGGCGCCCGGGACGTCCATGTACCCTCTCCCT |
| ATG18-YFPC-F | ATCACAATGGCCGGATCCATGGCGACTGCAACGCTAAAC |
| ATG18-YFPC-R | GCAGGCCGGGCGCCCGGGAGACTCATATGTCGAAGAAGA |
| MPS1-YFPC-F | ATCACAATGGCCGGATCCATGTCGGATCTCCAGGGCCGC |
| MPS1-YFPC-R | GCAGGCCGGGCGCCCGGGGCGGCCCTGGAGATCCGACAT |
| CDC28-YFPC-F | ATCACAATGGCCGGATCCATGGAAAACTACCAGAAGCTC |
| CDC28-YFPC-R | GCAGGCCGGGCGCCCGGGTCGCCTCGGGGGCGCTGGGGT |
| SWE1-YFPN-F | GTCAAAATGGTCGGATCCATGTCCTTCTCCAATTCGGGA |
| SWE1-YFPN-R | GGCGATGGAGCGCCCGGGAACGTCGGTCATCTCGACGTC |
| Primer used for Southern blot | |
| Probe-F | GCTCCGTAGTCGTAACATTCG |
| Probe-R | GACCTCAAGCCTGCCAATATC |
